# Drivers of Taxonomic Bias in Ecology and Evolution: Insights From Ethologists and Behavioural Ecologists

**DOI:** 10.1002/ece3.74460

**Published:** 2026-10-12

**Authors:** Pietro Pollo, Michael M. Kasumovic

**Affiliations:** ^1^ Evolution & Ecology Research Centre, School of Biological, Earth & Environmental Sciences University of New South Wales Kensington New South Wales Australia

**Keywords:** charismatic species, knowledge shortfall, model organism, model system, research bias, taxonomic chauvinism, taxonomic hyperfocus

## Abstract

Taxonomic bias, that is, the disproportionate attention given to some taxa relative to their diversity, continues to hinder innovation and the pursuit of generality in ecology and evolution. Yet, the factors shaping ecologists' and evolutionary biologists' choices of study organisms mostly remain a mystery. We propose a framework explaining researchers' taxonomic choices along three major axes, supported by evidence from a preregistered survey of 868 researchers' experiences. First, rational considerations, such as logistical ease and societal relevance, were associated with the choice of research organisms within major animal groups but rarely across them. Second, contextual factors, like the prominence of certain organisms within peer networks or early‐career exposure to specific taxa, were also associated with researchers' decisions about which organisms to study. Third, emotional factors, including taxonomic affinities, closely mirrored taxonomic patterns in the literature, indicating that they are a major driver of organism choice at broad taxonomic scales. Based on these findings, we suggest actions to mitigate taxonomic bias, including promoting (i) outreach initiatives featuring neglected taxa, (ii) taxonomically equitable education and (iii) taxonomically diverse research experiences.

## Graphical abstract

Taxonomic bias, the uneven research attention across organisms, limits generality in ecology and evolution, yet its causes remain unclear. Using a survey of 868 researchers, we show that rational, contextual and emotional factors jointly shape which organisms scientists choose to study. Our framework highlights mechanisms driving taxonomic bias and points to actionable strategies for promoting more taxonomically inclusive research.

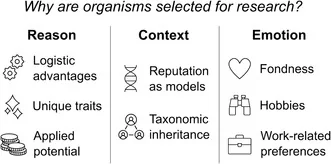

## 1 Introduction

One of the main goals of ecology and evolution is to comprehend biological processes and patterns across Earth's vast diversity of life. This goal is hindered when certain taxa receive disproportionally more attention than their diversity warrants, a pattern known as taxonomic bias or taxonomic chauvinism (Shine and Bonnet 2000; Bonnet et al. 2002). Taxonomic bias leads to the accumulation of knowledge on few organisms at the cost of the perpetuation of ignorance on all others, ultimately precluding theoretical generality (Bonnet et al. 2002; Dochtermann et al. 2026) and innovation (Ng et al. 2021; Broeckhoven and du Plessis 2022; Penick et al. 2022; Stuart‐Fox et al. 2023; Snell‐Rood and Smirnoff 2025). Unfortunately, taxonomic bias is widespread in ecology and evolution (Bonnet et al. 2002; Clark and May 2002; Seddon et al. 2005; Leather 2009; Stahlschmidt 2011; Zuk et al. 2014; Rosenthal et al. 2017; Titley et al. 2017; Troudet et al. 2017; Mammola, Riccardi, et al. 2020; Pollo et al. 2024; Pollo, Lagisz, et al. 2025; Guénard et al. 2025; Mizuno et al. 2025; Pollo and Kasumovic 2026). For instance, even though birds and mammals comprise < 1% of animal biodiversity (Zhang 2013a), these animals dominate both conservation (Seddon et al. 2005; Mammola, Riccardi, et al. 2020; Guénard et al. 2025) and research efforts in most subpockets of ecology and evolution (Bonnet et al. 2002; Clark and May 2002; Leather 2009; Stahlschmidt 2011; Rosenthal et al. 2017; Titley et al. 2017; Pollo et al. 2024; Pollo, Lagisz, et al. 2025; Mizuno et al. 2025; Pollo and Kasumovic 2026). Taxonomic bias also extends to finer taxonomic scales, with a handful of genera or species concentrating much of the research attention given to entire clades (Zuk et al. 2014). Investigations of the literature on specific groups of organisms (e.g., amphibians: da Silva et al. 2020; birds: Fischer et al. 2025; Murray et al. 2015; Yarwood et al. 2019; mammals: dos Santos et al. 2020; Tam et al. 2022; parasites: Simoncini et al. 2025; plants: Adamo et al. 2021; primates: Ellison et al. 2021; Chen et al. 2023; terrestrial vertebrates: Moura and Jetz 2021; see also Mammola et al. 2023) have shown that organisms' traits (e.g., size and colour), range and location can influence the amount of attention they receive, shedding some light on the drivers of taxonomic bias. However, such literature assessments present only indirect and taxon‐specific evidence regarding the origins of taxonomic bias, highlighting the absence of a general framework spanning multiple taxonomic levels and empirical support for it. In other words, why ecologists and evolutionary biologists choose the organisms they study largely remains a conundrum. Answering this question is challenging because it requires understanding the scientists that perform this research and what they seek in a study organism.

Science is built on prior research, so the popularity of an organism in an academic context may reinforce and amplify its own popularity, generating a feedback loop. This process partly stems from pragmatism: as more resources become available for an organism, further research on it becomes easier. However, there is also a subjective element to this process, as scientists may come to view certain organisms as ‘ideal’ study systems because of their established reputation rather than their intrinsic characteristics. This is essentially how so‐called model organisms arise (Leonelli and Ankeny 2012, 2013). While the intense use of such organisms has allowed important insights into biological mechanisms (Fields and Johnston 2005), ecologists and evolutionary biologists not necessarily interested in mechanistic questions have also frequently used model organisms in their research, leading to an unnecessary accumulation of knowledge on a few species (Zuk et al. 2014). Moreover, scientists' individual context may also influence the choice of research organisms. Researchers who continue working with the same taxa (often introduced by their supervisors early in their careers) enjoy a logistical advantage over those who attempt to study unfamiliar taxa. At the same time, fear of failure may also play a role in maintaining this focus, particularly as careers progress and the practical advantage of studying a familiar taxon over a new one diminishes. Ultimately, this form of taxonomic inheritance can perpetuate or even exacerbate existing taxonomic biases.

Scientists are often expected to be entirely rational in their research choices but, in reality, their emotions can play a major role in such decisions (Damasio 1994). For instance, researchers may be drawn to particular organisms out of fondness for them (Lorimer 2007). While inherent sensory biases may influence our fascination for organisms with certain traits (e.g., colourful; Langlois et al. 2022; Santangeli et al. 2023), other opinions about organisms, such as vertebrate animals being more valuable than invertebrate ones (Miralles et al. 2019; Possidónio et al. 2019), often arise from societal perceptions that develop from a young age. For example, children asked to draw natural landscapes are much more likely to depict vertebrates, particularly mammals and birds, than invertebrates (Strommen 1995; Snaddon et al. 2008). This skewed perception is then reinforced by the media (e.g., documentaries, Wei et al. 2024) and educational materials (e.g., textbooks, Gangwani and Landin 2018), helping to cement the idea that certain organisms are more important or interesting than others well into adulthood. It is thus plausible that the image that biologists have of nature, which is subjected to cultural factors and constructed before even joining academia, can influence their research choices. In fact, feelings of admiration and fascination towards certain taxonomic groups potentially entice early‐career biologists to work with these groups or may even encourage people to pursue a career in biology in the first place. By contrast, organisms that produce anxiety, fear or disgust (i.e., biophobias, Simaika and Samways 2010; Soga et al. 2023) may suffer from disinterest and avoidance from the general public and, consequently, have a lower research effort directed at them.

While some emotional links to taxa arise from society at large, others can be a product of habits and perceptions restricted to smaller social spheres. For instance, having hobbies that involve particular organisms (e.g., hunting or birding) may help construct positive feelings towards these organisms (Casola et al. 2026) and therefore skew research effort in their favour. Similarly, preferences for certain work‐related aspects, such as a strong inclination towards fieldwork (a sentiment shared by many biologists and reflected in the popularity of field courses; Fleischner et al. 2017; but see Soga and Gaston 2025), can influence researchers' taxonomic focus. This is because some organisms (e.g., many mammals and birds) can only be studied in the field, potentially attracting scientists that enjoy spending their time outdoors. Furthermore, although researchers may have predilections for certain organisms, their choice of study species can also be driven by other personal motivations. For example, researchers may be primarily motivated to investigate a particular topic (e.g., sexual selection), making the selection of a particular study organism less important to them. Understanding researchers' preferences and motivations then becomes pivotal to grasp drivers of taxonomic bias.

Here, we explored potential overarching drivers of taxonomic bias in ecology and evolution by directly surveying researchers (specifically ethologists and behavioural ecologists) about their experiences, preferences and perceptions regarding different taxa. We hypothesise that multiple rational, contextual and emotional factors are associated with the selection of research organisms (Figure 1), leading to a variety of predictions (Table 1; many preregistered in Pollo and Kasumovic 2025). After summarising our findings, we suggest interventions to reduce taxonomic bias in ecology and evolution, thereby increasing the likelihood of developing more generalisable theories in this field.

**FIGURE 1:**
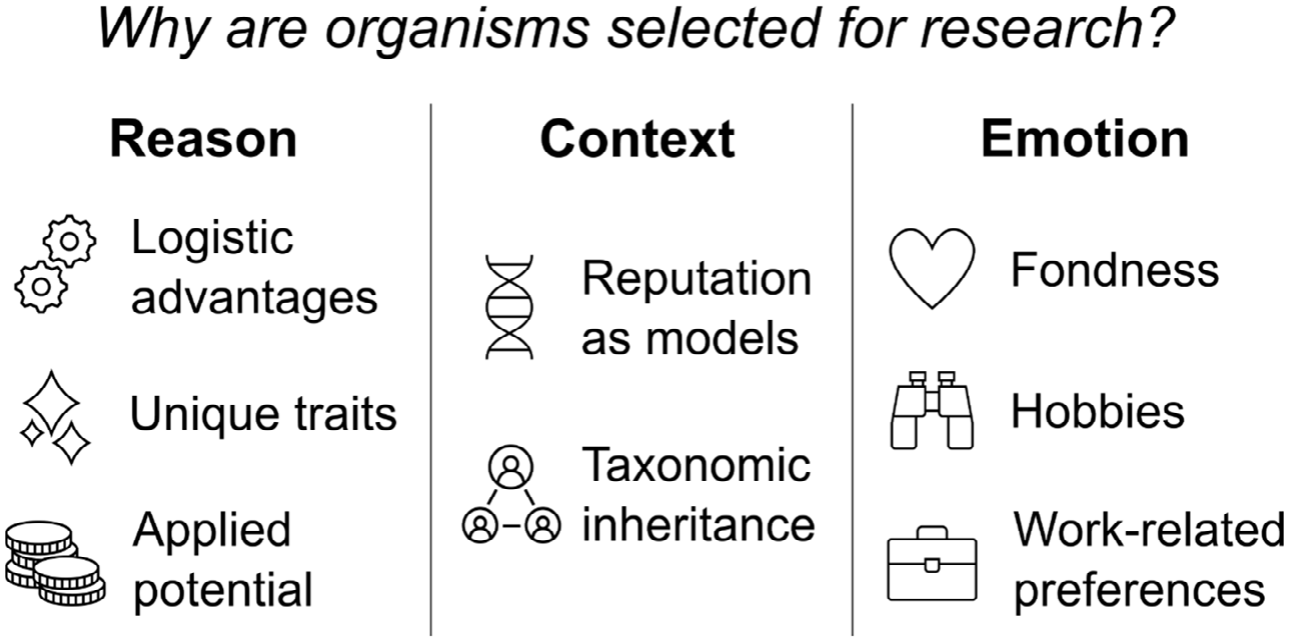
Factors hypothesised to be associated with the selection of research organisms in ecology and evolution under three axes: reason, context and emotion.

**TABLE 1:** Study predictions (some were preregistered in Pollo and Kasumovic 2025).

Axis	Factor	Prediction	Stated in preregistration	Support
Reason	Logistic advantages	Vertebrate animals, especially birds and mammals, are perceived as easier to work with in the field compared with other taxonomic groups.	Yes	Mixed: Figure 3A,B
		Vertebrate animals, especially birds and mammals, are perceived as harder to work with in the lab compared with other taxonomic groups.	Yes	Mixed: Figure 3C–E
		Within taxonomic groups, the more frequently a genus is researched, the greater the perception that it is easy to work with in the field.	No	Mixed: Tables S4 and S5
		Within taxonomic groups, the more frequently a genus is researched, the greater the perception that it is easy to work with in the lab.	No	Yes: Tables S4 and S5
	Unique traits	Within taxonomic groups, the more frequently a genus is researched, the greater the perception that it possesses a specific characteristic that makes it ideal to answer a question.	No	Yes: Tables S6 and S7
	Applied potential	Vertebrate animals, especially birds and mammals, are perceived as more economically important compared with other taxonomic groups.	No	Mixed: Figure 4B
		Vertebrate animals, especially birds and mammals, are perceived as more endangered or threatened in the wild compared with other taxonomic groups.	No	Mixed: Figure 4C
		Within taxonomic groups, the more frequently a genus is researched, the greater the perception that it is economically important.	No	Yes: Tables S6 and S7
		Within taxonomic groups, the more frequently a genus is researched, the greater the perception that it is endangered or threatened in the wild.	No	No: Tables S6 and S7
		Within taxonomic groups, the perception that an organism is economically important is positively associated with its use for applied research.	Yes	Yes: in‐text
		Within taxonomic groups, the perception that an organism is endangered or threatened in the wild is positively associated with its use for applied research.	No	Yes: in‐text
Context	Reputation as models	Within taxonomic groups, the more frequently a genus is researched, the greater the perception that it species is considered a model organism by the scientific community.	No	Yes: Tables S6 and S7
		The perception that a species is considered a model organism by the scientific community is positively associated with the perception of how extensive the literature on that species is.	Yes	Yes: in‐text
		The perception that a species is considered a model organism by the scientific community is positively associated with the perception of how easy it is to work with that species in the field.	Yes	Yes: in‐text
		The perception that a species is considered a model organism by the scientific community is positively associated with the perception of how easy it is to work with that species in the lab.	Yes	Yes: in‐text
	Taxonomic inheritance	An employer, supervisor, or collaborator is responsible for selecting the research species in most cases, except recently experienced species by individuals holding a permanent research position.	Yes	Yes: Figure 5
Emotion	Fondness	Vertebrate animals, especially birds and mammals, elicit more positive feelings than other taxonomic groups.	Yes	Yes: Figure S5
		Experience with a taxonomic group elicits positive feelings towards that group.	Yes	Yes: Figure 6A, Figure S5
		Experience with vertebrate animals, especially birds and mammals, is linked to a stronger motivation from taxon opposed to other factors.	Yes	No: Figure 6B
	Hobbies	Experience with birds is more often connected to cultural activities involving the observation of birds compared with experience with other organisms linked to observation of other organisms.	Yes	Mixed: Figure 4H
	Work‐related preferences	Experience with vertebrate animals, especially birds and mammals, is linked to a stronger preference for fieldwork (opposed to lab work).	No	Mixed: Figure 6B
Reason and emotion	Logistic advantages and work‐related preferences	Within taxonomic groups, preference for fieldwork (opposed to lab work) is positively associated with experience with species perceived as easy to work with in the field.	Yes	Yes: in‐text
		Within taxonomic groups, preference for fieldwork (opposed to lab work) is negatively associated with experience with species perceived as easy to work with in the lab.	Yes	Yes: in‐text

## 2 Material and Methods

Our methodology was described in our preregistration (Pollo and Kasumovic 2025), which is a research plan made prior to data collection and analysis (Wagenmakers et al. 2012). We highlight the deviations from our preregistration at the end of this section (see below). We report author contributions using MeRIT guidelines (Nakagawa et al. 2023) within this section (i.e., author initials clarify who did what) and the CRediT statement (McNutt et al. 2018) at the end of the manuscript.

### 2.1 Survey With Ethologists and Behavioural Ecologists

PP and MK formulated a survey with three sections: (1) demographics, (2) preferences, experiences and feelings and (3) perceptions regarding species used by participants for behavioural research (Appendix 1). For example, we asked participants from which organisms they collected behavioural data (Questions 12–15: Appendix 1), as well as how they felt towards distinct taxonomic groups on a spectrum ranging from negative (e.g., fear, anxiety) to positive (e.g., cuteness, fascination) feelings, with ambivalent feelings in between (Question 11: Appendix 1).

Our survey was available online from 27 May 2025 to 21 August 2025 to any person that desired to participate, was at least 18 years old, and had directly collected behavioural data from at least one non‐human organism that led to at least one research output of which they were an author (e.g., conference poster or presentation, preprint, peer‐reviewed publication, dissertation, thesis, book). No financial incentives were provided to participants, and anonymity was maintained by collecting only their survey responses. Ethical approval for this study was obtained from the University of New South Wales' Human Research Ethics Committee (reference number iRECS8512).

PP recruited participants by sharing the link to the survey on the social media site *Bluesky* (i.e., posting with his personal account), through email chains and via a targeted email list. This email list was obtained by extracting all emails associated with articles published in specific journals since 2015 or 2020 (Table S1). PP established these cut‐off years to reduce the number of extracted email addresses that were no longer valid. This process resulted in a list containing 20,818 unique email addresses, albeit 2879 were invalid (i.e., undeliverable) and another 708 generated automatic replies indicating that their users were unavailable (e.g., out of the office) at the time of delivery (PP did not contact them again). After accounting for these, 17,231 emails were considered to have most likely reached their intended recipients.

The survey was completed by 946 individuals, representing approximately 5.49% of the recruitment emails sent that likely reached recipients. However, an exact estimate of the recruitment success for our survey could not be calculated as the identity of participants was not collected and the total number of individuals that were reached and were eligible to participate in the survey was unknown. For instance, emails may have reached researchers that had never collected behavioural data.

PP carefully examined the responses obtained and excluded 77 of them from the dataset: 31 from participants that incorrectly answered the attention question in the survey (i.e., selected other options than agree for the question ‘please select the option agree’; Question 32: Appendix 1); seven responses with dubious veracity (a 99 years old participant, a participant that was 18 years old when they completed their PhD, and five participants with editorial experience despite not having a PhD); 37 contradictory responses (nine participants that worked with a greater number of taxonomic groups than of species, 28 participants that collected behavioural data of a species from a taxon they reported having no experience with); and three visibly duplicated responses (identical age, gender, country, year of PhD, species mentioned). As a result of these exclusions, 868 responses (91.75% of all responses obtained) were used for most analyses in the present study.

Our survey also asked participants to name the species they most and least recently collected behavioural data from (Question 14: Appendix 1). PP only considered these specific responses as valid for analyses involving them when they represented a genus or a species (common names were transformed to Latin names when possible). Consequently, PP excluded 86 answers out of the 1666 obtained (5.16%) that were too vague (e.g., ‘insect’ instead of a specific insect species or genus), contained multiple species, or were repeated by a participant (i.e., most and least recent species mentioned were the same). This means that we obtained 839 and 741 valid responses for species participants most and least recently worked with, respectively.

### 2.2 Statistical Analyses

First, to understand which factors were associated with the number of different species and taxonomic groups participants experienced (i.e., directly collected behavioural data from, leading to at least one research output in which they were an author), PP fitted two similar generalised linear models (GLMs), one with a negative binomial error distribution and the other with a Poisson error distribution. The response variable in the first GLM was the number of species that participants experienced, while the response variable in the second GLM was the number of taxonomic groups experienced (out of 11 options). Participant age (continuous), gender (men vs. women vs. other or unknown), editorial experience (binary: with vs. without), preference for fieldwork, and motivation from taxa served as predictor variables (the last two were treated as continuous variables even though participants provided responses in discrete increments of 10, ranging from 0 to 100). Additionally, the first GLM included the number of taxa experienced as a predictor variable, and the second GLM included the number of species experienced as a predictor variable.

Second, to examine participants' agreement (7‐point Likert scale) with statements about the species they most recently experienced (Questions 19–31: Appendix 1) and the species they least recently experienced (Questions 27 and 28: Appendix 1), PP fitted a distinct CLMM (i.e., mixed ordinal regression, statistical model used to analyse ordinal data) for each agreement item. In these CLMMs, the taxonomic group of species mentioned by participants served as the only predictor variable. The genus of the species mentioned was used as a random factor in all CLMMs, while participant ID also served as a random factor for the CLMMs related to Questions 27 and 28 (Appendix 1) (as there were up to two responses from each participant).

Third, PP fitted another set of CLMMs to again examine participants' agreement to particular statements, but this time within taxonomic groups. The specific statements analysed in these models were related to ease of working with the most recently experienced species (Questions 19–23: Appendix 1), its traits' idealness (Question 26: Appendix 1), economic importance (Question 29: Appendix 1), conservation status (Question 30: Appendix 1) and reputation as a model system (Question 24: Appendix 1). In these CLMMs, the number of participants mentioning the genus that the species belonged to was used as a predictor variable and the genus of the species mentioned was used as a random factor. Participants' preference for fieldwork was also added as a predictor variable when examining statements related to ease of working with the species mentioned (Questions 19–23: Appendix 1).

Fourth, to understand who drove participants' decisions on research organisms, PP fitted a generalised linear mixed model (GLMM) using a binomial error distribution on whether species mentioned by participants were recommended to them for research purposes by their supervisor vs. others (i.e., other people or themselves). Predictor variables in this GLMM included whether the species mentioned was the most or least recently experienced by them, and when participants first worked with the species they mentioned (factor with three levels: during/before their PhD, during postdoc or after obtaining a permanent position). Participant ID was then used as a random factor.

Fifth, to identify which factors were associated with participants' fondness for organisms, PP fitted cumulative linked models (CLMs) with a logit link on participants' feelings for each of the nine animal groups included in the survey (‘other invertebrates’ and ‘other organisms’ were not included in Question 11: Appendix 1). In addition to the variables used in GLMs aforementioned (i.e., age, gender, editorial experience, preference for fieldwork, motivation from taxa), these CLMs also included experience with the taxon in question as a predictor variable (binary: with vs. without). Furthermore, to compare fondness for distinct taxonomic groups across participants, PP fitted a cumulative linked mixed model (CLMM) on all nine responses of how participants felt towards distinct taxa together, using participant ID as random factor and taxonomic group as a predictor variable in these CLMMs.

Sixth, to examine which factors were linked to participants' taxonomic experiences, PP fitted a separate GLM for each of the 11 taxonomic groups included in the survey (amphibians, arachnids, birds, crustaceans, fish, insects, mammals, molluscs, other invertebrates, other organisms and reptiles), all using a binomial error distribution. The response variable in each of these GLMs indicated whether a participant had experience with the taxon in question. All these GLMs included the same predictors aforementioned (age, gender, editorial experience, preference for fieldwork and motivation from taxa) and, except for ‘other invertebrates’ and ‘other organisms’, participants' feelings for the taxon in question (factor with up to seven levels).

In all models described above, continuous predictor variables were scaled by subtracting the mean from each value and then dividing the result by the standard deviation. PP identified predictor variables associated with response variables tested using model selection. He selected models whose Akaike Information Criterion corrected for small sample sizes (AICc) was within two units of the model with the lowest AICc. He then fitted simplified versions of these models by including only predictor variables that appeared in all selected models, meaning that he assumed that only these variables were associated with response variables. Lastly, PP used Spearman's correlations to verify pairwise relationships between variables that were on a 7‐point Likert scale. Unless otherwise stated, means were presented with standard error.

PP conducted all analyses using R (R Core Team 2025) v. 4.5.1. GLMMs were fitted using the package *glmmTMB* (Brooks et al. 2017; McGillycuddy et al. 2025) v. 1.1.12 and ordinal regressions (CLMs and CLMMs) were conducted using the package *ordinal* (Christensen 2023) v. 2025.12.29. PP verified GLMMs' assumptions using the package *DHARMa* (Hartig 2024) v. 0.4.7. and computed model predictions using the package *emmeans* (Lenth 2025) v. 1.11.2.8. PP used the package *MuMIn* (Bartoń 2025) v. 1.48.11 to obtain and compare all possible candidate models from complex models.

### 2.3 Deviations From the Preregistration

We adhered to our preregistration (Pollo and Kasumovic 2025) with some deviations. For instance, PP and MK used part of the original plan to generate a separate study assessing existing patterns of taxonomic bias in ecology and evolution (Pollo and Kasumovic 2026). Importantly, not all predictions tested in this study were preregistered (see Table 1) and full specifications of statistical models conducted were not provided in the preregistration. Furthermore, PP initially planned to assess relationships between variables using a 7‐point Likert scale with polychoric multivariate analyses. However, because applying this method on several pairs of variables from the dataset often violated statistical assumptions related to this method, PP opted to use Spearman's correlations instead.

## 3 Results

### 3.1 Participant Profile

Participants in our survey (*N* = 868) were, on average, 42.89 ± 11.56 years old (mean ± SD; Question 2: Appendix 1). 52.76% of them identified as men, 44.82% as women, 1.73% as another gender identity (e.g., gender‐fluid, non‐binary), and 0.69% did not disclose their gender identity (Question 3: Appendix 1). We asked each participant to name the country where they spent most of their life before and after reaching adulthood (18 years old; Questions 4 and 5: Appendix 1), to which we obtained 68 unique countries before age 18 and 59 after age 18 (Figure S1), with 80.65% of participants selecting the same country for both periods. Most participants (89.97%) held a PhD degree (Question 6: Appendix 1) and more than a third of all participants (36.52%) had editorial experience (Question 8: Appendix 1).

### 3.2 Participant Preferences and Experiences

On a scale from 0% to 100%, we asked participants to determine how they would split their time collecting behavioural data between the field and the lab based purely on personal enjoyment (hereby *preference for fieldwork*; Question 9: Appendix 1), and how much their desire to work with certain taxa drove their behavioural research interests compared with other motivations (hereby *motivation from taxa*; Question 10: Appendix 1). On average, preference for fieldwork was 59.27% ± 1.02% and motivation from taxa was 50.76% ± 0.96%, but responses to these questions varied across participants (Figure S2A,B).

Participants experienced (i.e., directly collected behavioural data that led to at least one research output in which they were an author), on average, 9.48 ± 0.45 species (Figure S2C; Question 12: Appendix 1). However, this estimate would be higher if we had not (unintentionally) set a maximum of 80 in the survey platform for this question (16 participants answered this maximum number; Figure S2C). From the 11 taxonomic groups listed in our survey as options (amphibians, arachnids, birds, crustaceans, fish, insects, mammals, molluscs, other invertebrates, other organisms and reptiles), participants experienced, on average, 2.1 ± 0.04 of them (Figure S2D; Question 13: Appendix 1). Participants that experienced more species, on average, also experienced more taxonomic groups (Figure 2; Table S2). Age and motivation from taxa were associated with both the number of species and the number of taxonomic groups experienced, while gender and editorial experience were only related to the number of species (Figure 2A,C; Tables S2 and S3). By contrast, preference for fieldwork was unrelated to the number of species or taxonomic groups experienced (Figure 2; Tables S2 and S3).

**FIGURE 2:**
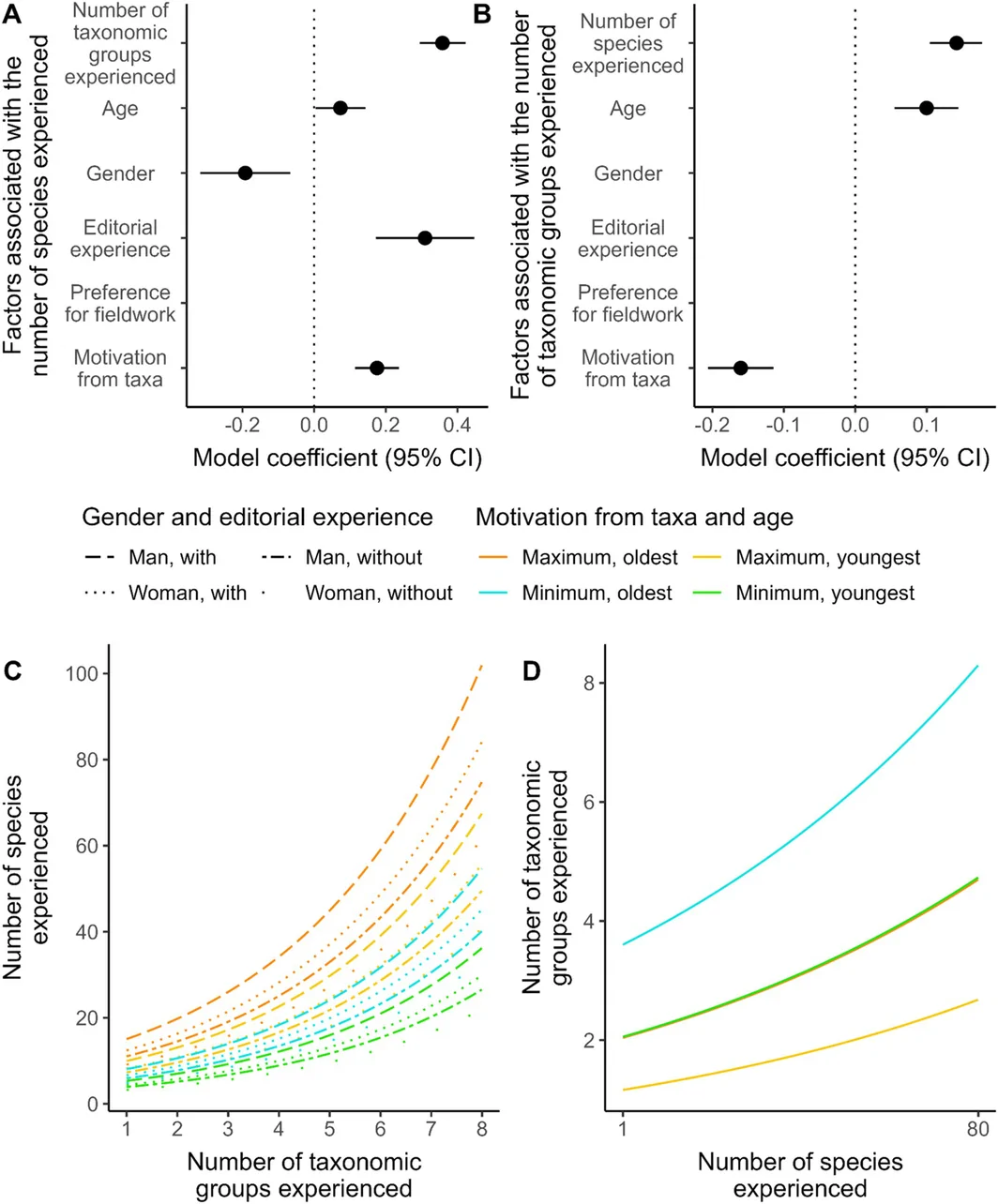
Factors associated with participants' experiences. Top panels show coefficient values for factors associated with the number of species (A) and taxonomic groups (B) that participants experienced (i.e., directly collected behavioural data from, leading to at least one research output of which they were an author). Gender refers to the comparison of women vs. men (comparisons with other or unknown gender identities are not shown). Note that only coefficients from factors included in all selected models are shown. Bottom panels show the number of species (C) and taxonomic groups (D) that participants worked with predicted by selected models. Maximum and minimum motivation from taxa refer to 100% and 0% of this variable (respectively), while oldest and youngest represent 82 and 21 years old individuals (respectively).

When considering all taxonomic groups participants experienced, birds and mammals were the taxonomic groups most frequently disclosed (each by 42.58% of participants), followed by insects (35.21%) and fish (24.05%; Figure S3). A similar pattern emerged when considering the taxonomic group of participants' most and least recently experienced species, with mammals and birds representing more than half of all responses (Figure S5A). We also noted that many researchers shared the same species mentioned. For example, *Drosophila melanogaster* , *Rattus norvegicus* and *Parus major* were mentioned by 22, 21 and 17 participants, respectively. In fact, only 65.29% and 49.68% of all responses for specific organisms experienced by participants represented unique species and genera, respectively. The most popular taxonomic groups, especially mammals, were the ones with the relatively fewest unique species (Figure S5B) and genera (Figure S5C).

### 3.3 Rational Factors Behind Taxonomic Choices

We found that the most popular organisms (i.e., birds and mammals) were rarely perceived as being easier to work with than others (Figure 3; Questions 19–23: Appendix 1). Furthermore, within taxonomic groups, the number of times a genus was mentioned by distinct participants (as the one they most recently worked with) was positively related to all aspects aforementioned regarding how easy they are to work with (Tables S4 and S5). Lastly, after controlling for the genus and the taxonomic group of the species mentioned by participants, a stronger preference for fieldwork was associated with perceiving the species most recently experienced as easier to observe in the field (though not necessarily easier to find there), and harder to buy, rear or observe in the laboratory (Tables S4 and S5).

**FIGURE 3:**
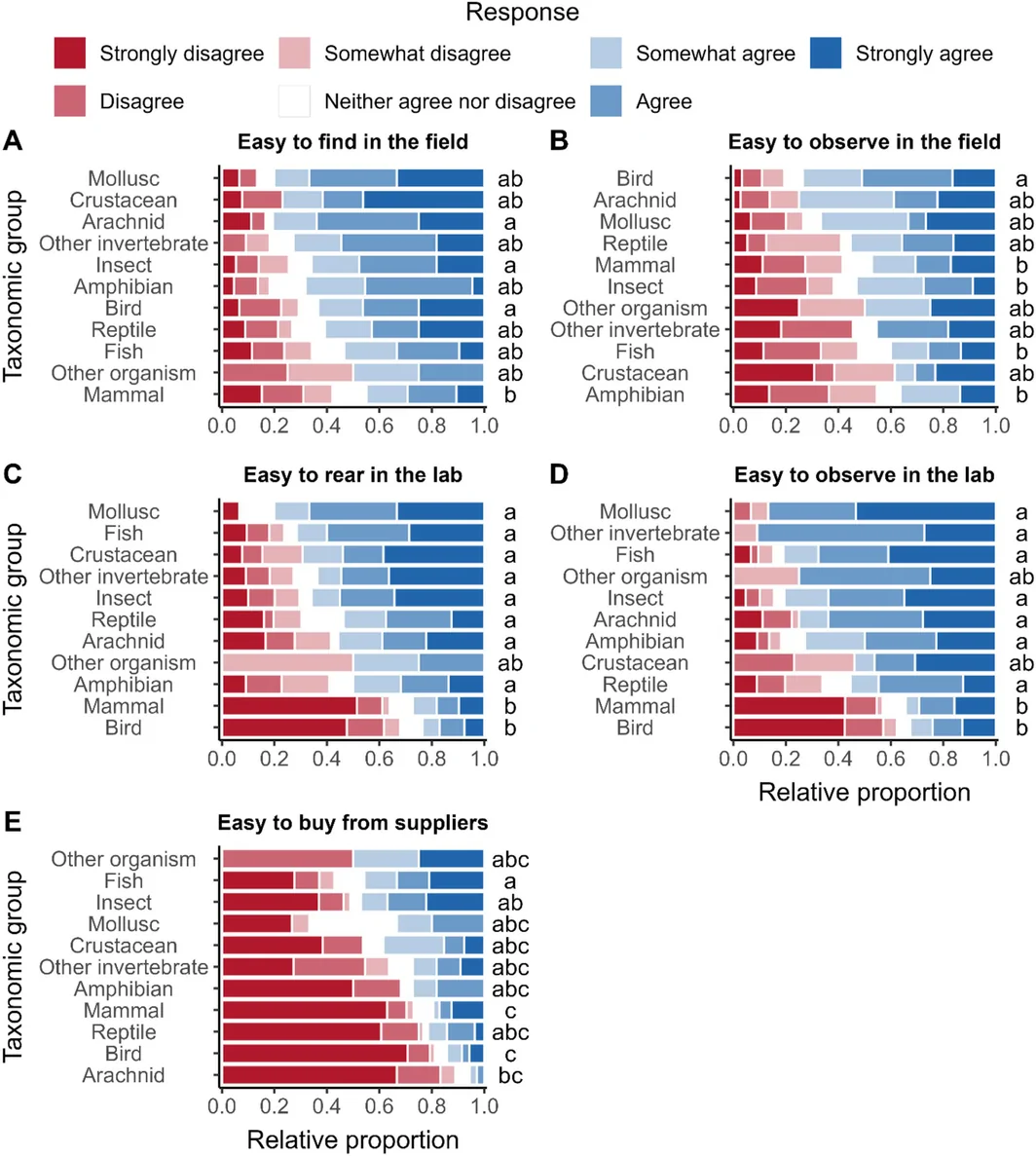
Participants' agreement with statements on the ease to, regarding the organism they most recently worked with, locate in the field (A), observe in the field (B), rear in the lab (C), observe in the lab (D) or buy from suppliers (E). Distinct letters on the right side of bars represent statistical differences between taxonomic groups within that panel (*z*‐values with *p* < 0.05 for all pairwise comparisons).

We did not detect any differences among taxonomic groups in whether the participants' most recently experienced species possessed a characteristic that made it an ideal species to answer a question they were interested in (Figure 4A; Question 26: Appendix 1). However, within taxonomic groups, the number of times a genus was mentioned by distinct participants as the one they most recently worked with was positively related to the perception of idealness of the genus in question (Tables S6 and S7).

**FIGURE 4:**
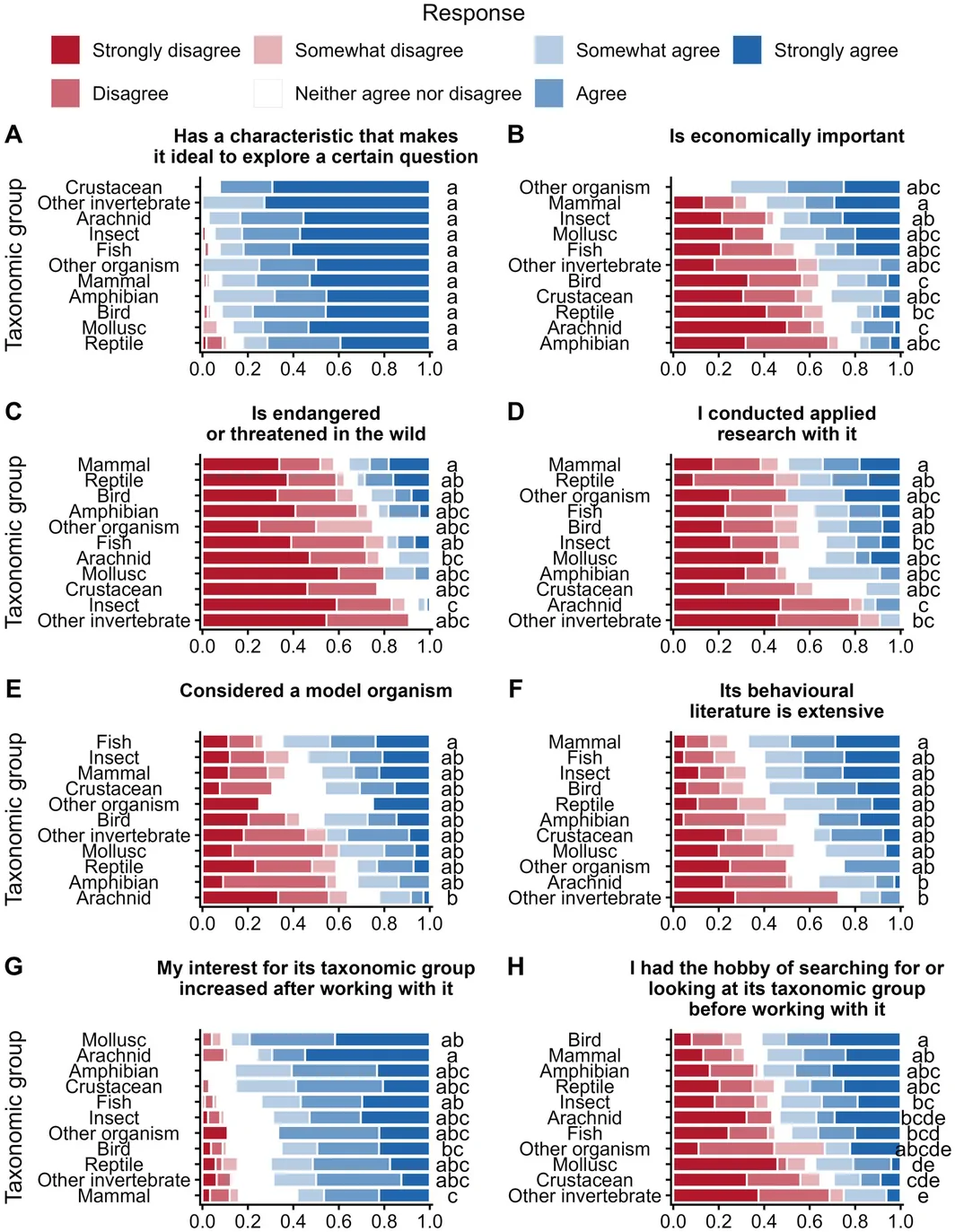
Participants' agreement with various statements related to one or two organisms they worked with (panel A: possession of a specific characteristic; panel B: economic importance; panel C: conservation status; panel D: applied research use; panel E: status as a model organism; panel F: literature extent; panel G: interest after research; panel H: related hobby). Distinct letters on the right side of bars represent statistical differences between taxonomic groups within that panel (*z*‐values with *p* < 0.05 for all pairwise comparisons).

We observed that participants' perceived applied potential (e.g., economic importance and conservation status) of their most recently experienced species was greater if it belonged to certain taxonomic groups (especially mammals; Figure 4B–D; Questions 29–31: Appendix 1). Within taxonomic groups, the number of times a genus was mentioned by distinct participants as the one they most recently worked with was positively related to the perceived economic importance of that genus but negatively related to the perceived endangered or threatened status in the wild of that genus (Tables S6 and S7). Participants' agreement with the statement that the research they conducted with the most recently experienced species mentioned was applied was moderately linked to their perception that the species is economically important (*r*_*s*_ = 0.331) and weakly associated with their perception that the species is endangered or threatened in the wild (*r*_*s*_ = 0.241).

### 3.4 Contextual Factors Behind Taxonomic Choices

Fish were more often perceived as model organisms than arachnids (Figure 4E; Question 24: Appendix 1), while mammals were more commonly associated with an extensive behavioural literature than arachnids and unnamed invertebrates (Figure 4F; Question 25: Appendix 1). Within taxonomic groups, the number of times a genus was mentioned by distinct participants as the one they most recently worked with was positively related to the perception that the genus is considered a model organism by the scientific community (Tables S6 and S7). Moreover, we found that participants' perception that the species they most recently experienced is a model organism was positively related to multiple other attitudes about the same species with varying magnitudes: (1) strongly related to the impression that the behavioural literature on the species is extensive (*r*_*s*_ = 0.643); (2) moderately related to the impression that the species is easy to buy (*r*_*s*_ = 0.456), rear (*r*_*s*_ = 0.404), and observe in the laboratory (*r*_*s*_ = 0.401); (3) weakly related to the impression that the species is easy to find (*r*_*s*_ = 0.184) and observe in the field (*r*_*s*_ = 0.119). Notably, the species that participants most and least recently experienced were often suggested by their supervisors as a research system, except when participants already held a permanent position (Figure 5; Table S8; Questions 17 and 18: Appendix 1).

**FIGURE 5:**
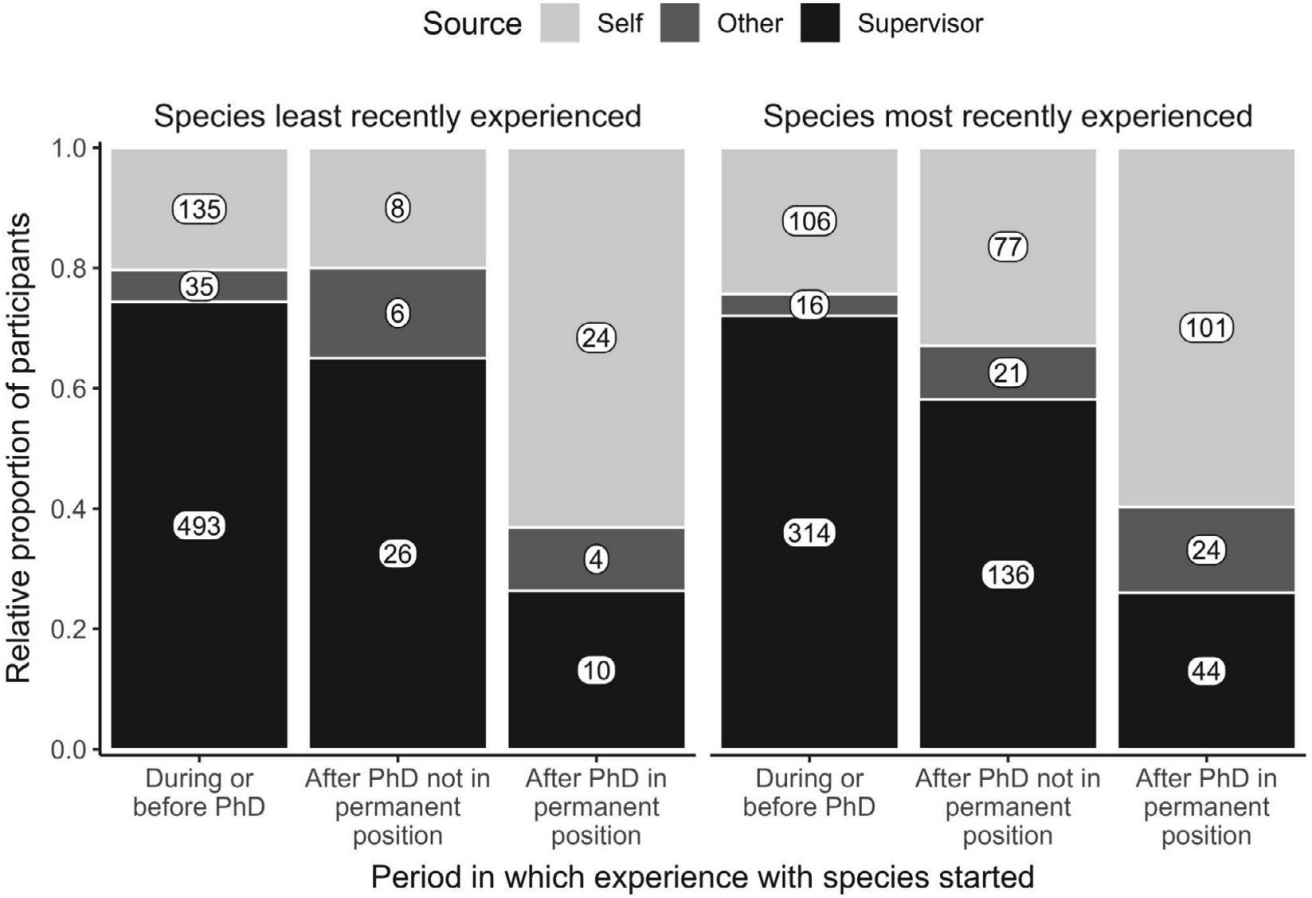
How and when participants started working with certain species. Numbers within bars represent the number of participants within that category.

### 3.5 Emotional Factors Behind Taxonomic Choices

All animal groups mostly elicited positive feelings from participants, although some (mainly vertebrates) more than others (Figure S5; Question 11: Appendix 1). On average, participants declared more favourable perceptions of taxa they experienced than of other taxa (*β* = 2.52 ± 0.07, *z* = 33.91, *p* < 0.001; Figure 6A, Figure S5B,C; Table S9), although multiple other factors were also associated with how participants felt about certain taxa (Figure 6A; Table S9). Most participants disclosed that their interest for the taxonomic group of species they worked with grew after their experience (Figure 4G; Question 28: Appendix 1).

**FIGURE 6:**
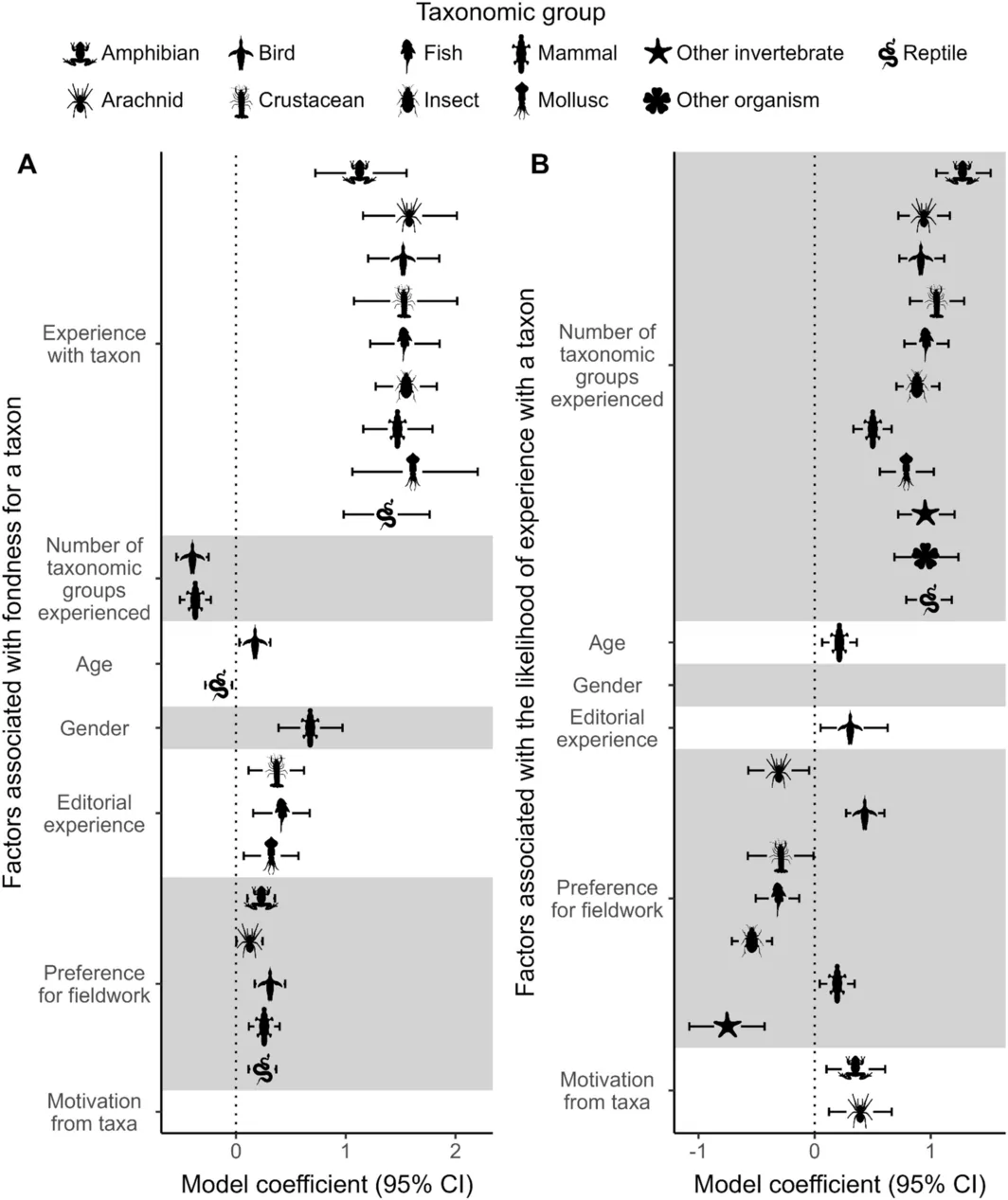
Factors associated with fondness for (A) and experience (i.e., direct collection of behavioural data that led to at least one research output) with (B) a given taxonomic group. Gender refers to the comparison of women vs. men (comparisons with other or unknown gender identities are not shown). Fondness for a taxon was also included as a predictor in models exploring the factors associated with the likelihood of experience with that taxon (B), but coefficients are not shown. Background colours highlight coefficients associated with different factors.

Participants that mentioned a bird as their most or least recently experienced species were more likely to have engaged in birding than participants that mentioned an invertebrate were to have engaged in equivalent hobbies with invertebrates (Figure 4H; Question 27: Appendix 1). Furthermore, we found several associations between experience with certain taxa and other factors, especially preference for fieldwork (Figure 6B; Table S10).

## 4 Discussion

In the present study, we hypothesised that several factors (stemming from reason, context or emotion, Figure 1) are related to the selection of research organisms and thus drivers of taxonomic bias. The survey we conducted with ethologists and behavioural ecologists provides evidence in favour of this framework, although in some cases, only at certain taxonomic levels. Below we discuss each of our main findings in detail, but see Appendix 2 for the discussion of our secondary results.

The taxonomic experiences of participants in our survey mirrored patterns of taxonomic bias present in the animal behaviour literature (Rosenthal et al. 2017; Pollo and Kasumovic 2026). For instance, birds and mammals were the taxonomic groups participants in our survey were most likely to have worked with (Figure S3; Figure S4A). Conversely, apart from insects, participants rarely used invertebrates as research organisms (Figure S3; Figure S4A) despite their immense diversity (Zhang 2013a; Eisenhauer and Hines 2021) and the fact that some of them are widely considered model organisms (e.g., the nematode *Caenorhabditis elegans* ). Moreover, we observed that certain organisms (at the species or genus level) concentrated much of the attention given to their taxonomic group (Figure S4B,C), reiterating that taxonomic bias also occurs at fine taxonomic levels (Zuk et al. 2014). Note, however, that the popularity of some organisms may be a spillover from the popularity of the organisms they are associated with, such as parasites of charismatic mammals (Simoncini et al. 2025). For instance, ticks are mentioned in 20.1% of ecology and evolution papers that mention arachnids (Pollo and Kasumovic 2026) despite representing 0.08% of arachnid species (Zhang 2013b).

The popularity of taxonomic groups among participants in our survey was rarely associated with how easy they are to access and work with, showing that organisms' logistic aspects do not appear to be associated with their research use at high taxonomic levels. For example, with some exceptions, birds and (especially) mammals were considered as equally or more difficult to find, observe, rear, or buy than other taxonomic groups (Figure 3). As a counterpoint, molluscs, which were rarely used by surveyed scientists for research purposes (only 5.88% of participants worked with them; Figure S3), were largely perceived as easy to work with both in the field and in the lab (Figure 3). These results are not surprising as the necessary fieldwork duration to collect data from overlooked taxa (beetles and moths) is usually shorter than from popular taxa (mammals and birds) (Pawar 2003). Nonetheless, within taxonomic groups, genera mentioned more frequently by participants tended to be perceived as easier to work with. This suggests that organisms with logistic advantages may be selected over other closely related taxa, a result that is congruent with studies investigating literature patterns related to specific taxonomic groups (Yarwood et al. 2019; dos Santos et al. 2020; da Silva et al. 2020; Ellison et al. 2021; Tam et al. 2022; Chen et al. 2023; Fischer et al. 2025).

We hypothesised that researchers also consider the possible applications of organisms to society, such as their economic relevance or conservation status, when deciding which research to pursue. We found that insects and mammals were deemed more economically important than arachnids and birds (mammals were also considered more economically important than reptiles). By contrast, insects were less frequently deemed threatened or endangered in the wild than most taxonomic groups (including mammals, which were also more frequently deemed endangered than arachnids). This finding is worrisome as invertebrates are under severe threat (Cardoso et al. 2020) yet their conservation remains underfunded (Guénard et al. 2025). Invertebrates are also underrepresented in biodiversity databases (e.g., IUCN's red list; Cardoso et al. 2012) as most biodiversity assessments focus on birds and mammals, making their decline and extinction unnoticed (Régnier et al. 2015). This represents a striking example of the taxonomic bias vortex, in which lack of data generates the impression that no problem exists, leading to even less attention to the issue. Participants were also more likely to state that they had conducted applied research with species they deemed more economically important or more endangered in the wild. Strangely, the likelihood of participants conducting applied research was greater for mammals than for arachnids and insects (Figure 4D) despite insects being perceived as economically important as mammals (Figure 4B), and despite the fact that invertebrates provide multiple ecosystem services (Eisenhauer and Hines 2021; Cardoso et al. 2025). Within taxa, the number of participants working with a genus was positively related to its perception as economically important, but negatively related to its perception as endangered or threatened in the wild. Our results indicate that organisms' aspects connected to their explicit usefulness to society can be involved in researchers' taxonomic decisions, especially at finer taxonomic scales, but this influence seems to be weak, at least among ethologists and/or behavioural ecologists.

In our survey, participants frequently deemed several species as model organisms (e.g., barn swallows, crab‐eating macaques) despite them not being officially listed as such (by USA's National Institute of Health; NIH 2025). Participants' agreement that an organism is a model system was positively related to the number of distinct participants mentioning the genus of that organism, indicating that this term is loosely applied to commonly studied species in a certain context (in our case, behavioural research). Species considered model organisms by participants in our survey were also more likely to be deemed easy to work with in the lab than in the field, confirming the connection of this term with experimental and laboratory work (Leonelli and Ankeny 2012, 2013; Dietrich et al. 2014; Matthews and Vosshall 2020). Despite this, the only difference across taxonomic groups we found was between fish and arachnids (Figure 4E), which is surprising given that they are perceived as similarly accessible to work in the laboratory (Figure 3C,D).

Given that the model organism label propulses taxonomic bias by rewarding and promoting species that have acquired an arbitrary status among researchers, we argue that this term should be abolished in most spheres of ecology and evolution. Moreover, even though species considered model organisms have generated breakthroughs in science (Fields and Johnston 2005), they cannot replace the hidden gems that biodiversity has to offer (e.g., bioinspired designs and materials; Ortiz and Boyce 2008). We should thus facilitate research with different species, both in the field and in the laboratory, instead of re‐using the same ones as a cheap trick to bypass a broken funding system. Recent technologies (e.g., drones, minute tracking devices; Farine et al. 2024) and data sources (e.g., citizen science; Jarić et al. 2020; Niemiller et al. 2021; Mason et al. 2025) can certainly help with this, allowing ecology and evolution to reach its full potential.

Precedence effects can also occur at the individual level. Most participants in our survey mentioned working, in multiple stages of their career, with species that were suggested by their supervisors, albeit this pattern was weakened after participants obtained a permanent position (Figure 5). This means that the taxonomic experiences of early career researchers (ECRs) are largely influenced by late‐career researchers (LCRs) they work with. ECRs often interact with few LCRs who can act as their mentors and/or supervisors (e.g., those employed by the university in which they study), representing a major bottleneck for ECRs' possible experiences, especially if LCRs only accept supervising research projects with a specific taxon. Because few LCRs work with neglected taxonomic groups, ECRs desiring to work with such groups are less likely to find supervisors who can support their interests. ECRs that manage to work with their predilected taxonomic group or those that do not have taxonomic preferences are then mostly bound to the specific organism suggested by the LCR supervising them. These processes can then dramatically reinforce existing taxonomic biases if researchers continue to work with the same taxonomic group or species throughout their career. Our findings suggest that researchers commonly take this route, stagnating or even amplifying taxonomic biases at multiple taxonomic levels. Fortunately, there are several tools that can help researchers to diversify their taxonomic experiences. For example, citizen science data (e.g., iNaturalist) can show species that are abundant in a given researcher's area, and enthusiasts (e.g., photographers, social media influencers) and other researchers (e.g., taxonomists) may be happy to suggest species to study and ways of investigating them in the field and in the lab. To ameliorate this precedence effect, we recommend that (1) institutions consider the diversity of taxonomic experiences of LCRs when recruiting them, (2) LCRs adopt a flexible approach, supporting and motivating ECRs to engage with diverse taxa, (3) ECRs search for distinct sources of information to expand their experiences. This effort could be further supported by universities and funding organisations through inclusion criteria that favour neglected taxa in hiring and grant assessments, encouraging both LCRs and ECRs to engage with a broader range of study organisms.

Researchers appear to let their emotions lead their choice of research organisms: stronger affection for a taxonomic group corresponded with a higher likelihood of working with it among participants in our survey (Figure 6B; Figure S5). Given that the most popular taxonomic groups (e.g., birds and mammals) overwhelmingly elicited positive feelings from researchers (including those that never worked with them; Figure S5), we argue that positive views of a taxon make researchers more likely to work with that taxon. Importantly, the patterns of fondness for distinct taxonomic groups we observed from surveyed researchers echoes values present in society, in which vertebrates are generally more well perceived than invertebrates (Miralles et al. 2019; Possidónio et al. 2019). We also found that participants' experiences with an organism stimulated more positive perceptions of its taxonomic group (Figure 4G), whereas this effect was stronger for arachnids than for birds and mammals, and also stronger for fish than for mammals. The relationship between fondness and experience appears to be reciprocal: appreciation of a taxonomic group increases the likelihood of working with it, while experience with a taxonomic group enhances appreciation of it (Figure 4G). This self‐reinforcing cycle resembles a positive counterpart of the conceptual framework for biophobia (Soga et al. 2023). In other words, our findings indicate that familiarity generates affinity, so taxonomic bias may be self‐perpetuating as researchers gravitate towards organisms they already like and, in doing so, become even more attached to them, while unfamiliar taxa remain avoided. Outreach programs are thus crucial to promote awareness and appreciation of neglected taxonomic groups among the general public, countering fear‐based narratives commonly perpetuated by the media that only aggravate this dire situation (Mammola, Nanni, et al. 2020; Mammola et al. 2022; Soga et al. 2023). Moreover, endorsing diverse taxonomic experiences in teaching and supervision within academia may also provide opportunities for early‐career researchers to develop interest in often overlooked organisms, helping shift the taxonomically biased *status quo* in ecology and evolution.

Specific activities and preferences also appear to be connected to researchers' taxonomic choices. For example, participants were more likely to have engaged in birding prior to working with a bird species than of having engaged in a similar hobby with an invertebrate taxon before having an experience with that taxon (Figure 4H). This bolsters the idea that positive exposure to a taxonomic group elicits individuals to work with that group, highlighting that encouraging activities involving neglected taxonomic groups (e.g., bugwatching; Eaton 2025) can generate research interest in the organisms involved. Furthermore, we found that surveyed researchers were diverse in their preference for fieldwork over lab work, ranging from complete to no preference (Figure S2A). Greater preference for fieldwork was not associated with how many species or taxonomic groups a surveyed researcher worked with (Figure 2), but it was positively related to greater appreciation of arachnids and vertebrates other than fish (Figure 6A). Yet, greater preference for fieldwork was only positively related to the likelihood of working with birds and mammals, but not with amphibians and reptiles, and negatively related to the likelihood of working with fish and invertebrates other than molluscs (Figure 6B). These findings suggest that professional preferences may influence scientists' fondness for certain taxa and their choices of research organisms (sometimes in conflicting ways).

Researchers also appear to vary in what motivates them, as some participants in our survey were exclusively driven by specific taxa while others were fully motivated by other factors (e.g., particular topics, professional prospects) (Figure S2B). The more participants were motivated by taxa, the more species but the fewer taxonomic groups they worked with (Figure 2). This suggests that researchers primarily motivated by taxa are open to expanding their experience towards other (preferably neglected) species from the same taxonomic group, while their counterparts can be more easily inspired to diversify their experiences at a higher taxonomic level. Additionally, although motivation from taxa was not associated with fondness for any taxonomic group (Figure 6A), the fact that it was positively related to the likelihood of working with amphibians and arachnids (Figure 6B) indicates that these two taxonomic groups are more likely to receive attention from researchers specifically interested in studying only them or a few other taxa.

Taxonomic bias generates a skewed perspective of natural processes and patterns, but it is only one of many biases affecting the study of ecology and evolution (Winder et al. 2025; Wascher et al. 2026). For example, biases in our perceptions of the sexes likely produce stereotypic portrayals of them (Pollo and Kasumovic 2022) (e.g., coy females and eager males), which are common in the literature (Green and Madjidian 2011; Ah‐King 2022a, 2022b; Spaulding and Fuselier 2023). Misrepresentations of taxa, sexes, and other biological aspects curb the power of studies attempting to draw broad conclusions about nature and ultimately impact the success of conservation projects (Gissi et al. 2024). On one hand, gaps in the available data, if properly detected, only allow part of the puzzle to be revealed. For example, lack of data on sexual signals expressed by females and animals other than birds precludes a holistic understanding of the evolution of these traits (Pollo, Lagisz, et al. 2025). Studies pointing out gaps in the literature can then direct researchers to collect data that is urgently needed (Pollo, Martinig, et al. 2025). However, researchers may infer erroneous conclusions when they fail to recognise that the data they analyse are biased. This is apparently a frequent issue in meta‐analyses related to animal behaviour, in which the underrepresentation of invertebrates leads to improper generalisations (Dochtermann et al. 2026). Awareness of biases in science is essential for their detection and mitigation (Winder et al. 2025) and thus an important first step. However, we argue that we need to go beyond theoretical discussions by implementing actions that minimise the existing biases in ecology and evolution. Similar to pleas made by researchers combating biases in how sexes are represented in science (Ah‐King 2013; Hughes 2022; Zemenick et al. 2022), we advocate for a more taxonomically equitable education (as early as childcare) and data collection. We hope that our recommendations throughout the present study can then influence ecologists and evolutionary biologists to strive for such endeavour (but see also Pollo and Kasumovic 2026).

## Author Contributions

**Pietro Pollo:** conceptualization (lead), data curation (lead), formal analysis (lead), investigation (lead), methodology (lead), project administration (lead), visualization (lead), writing – original draft (lead), writing – review and editing (lead). **Michael M. Kasumovic:** methodology (supporting), writing – review and editing (supporting).

## Conflicts of Interest

The authors declare no conflicts of interest.

## Supporting information


**Figure S1:** Participants' country. Country in which participants spent most of their life before (A) and after (B) being 18 years old. No participants mentioned countries or regions in grey.
**Figure S2:** Participants' preferences, motivations and overall experiences. Proportion of participants expressing their preference for fieldwork over lab work (A) and their motivation from taxa over other factors (B). Proportion of participants that experienced (i.e., directly collected behavioural data from, leading to at least one research output of which they were an author) distinct numbers of species (C) and of taxonomic groups (D).
**Figure S3:** Taxonomic groups that participants experienced (i.e., directly collected behavioural data from, leading to at least one research output of which they were an author). Note that each participant could experience multiple taxa.
**Figure S4:** Taxonomic groups of specific species that participants most and least recently experienced (i.e., directly collected behavioural data from, leading to at least one research output of which they were an author).
**Figure S5:** Participants' feelings towards distinct animal groups. Responses from all participants together (A) or by experience with the taxon in question (B: without, C: with). In panel A, distinct letters on the right‐side of bars represent statistical differences between taxonomic groups within that panel (*z*‐values with *p* < 0.05 for all pairwise comparisons). In Panels B and C, numbers on the right‐side of bars represent the number of participants for each taxon in the subset presented, with a total of 868 for each taxon.
**Table S1:** Journals used in our study to retrieve researchers' emails. ISSN stands for International Standard Serial Number.
**Table S2:** Model comparison regarding the number of species experienced by participants in our survey. Several possible predictor variables were tested (see main text for details). ‘+’ represents that the predictor variable was included in models shown. AICc and W stand for models' Akaike information criterion for small sample sizes and their weight, respectively. Only selected models (i.e., ΔAICc < 2) are shown.
**Table S3:** Model comparison regarding the number of taxonomic groups experienced by participants in our survey. Several possible predictor variables were tested (see main text for details). ‘+’ represents that the predictor variable was included in models shown. AICc and W stand for models' Akaike information criterion for small sample sizes and their weight, respectively. Only selected models (i.e., ΔAICc < 2) are shown.
**Table S4:** Model comparison regarding five statements (response variables) about the ease to work with the most recent species experienced by participants in our survey. ‘+’ represents that the predictor variable was included in models shown. AICc and W stand for models' Akaike information criterion for small sample sizes and their weight, respectively.
**Table S5:** Coefficients from models selected regarding five statements (response variables) about the ease to work with the most recent species experienced by participants in our survey (see also Table S4).
**Table S6:** Model comparison regarding four statements (response variables) about the most recent species experienced by participants in our survey. ‘+’ represents that the predictor variable was included in models shown. AICc and W stand for models' Akaike information criterion for small sample sizes and their weight, respectively.
**Table S7:** Coefficients from models selected regarding four statements (response variables) about the most recent species experienced by participants in our survey (see also Table S6).
**Table S8:** Model comparison regarding whether the species mentioned by participants in our survey were suggested by a supervisor (vs. other people or themselves). Two possible predictor variables were tested (see main text for details). ‘+’ represents that the predictor variable was included in models shown. AICc and W stand for models' Akaike information criterion for small sample sizes and their weight, respectively.
**Table S9:** Model comparison regarding the fondness by participants in our survey for each taxonomic group. Several possible predictor variables were tested (see main text for details). ‘+’ represents that the predictor variable was included in models shown. AICc and W stand for models' Akaike information criterion for small sample sizes and their weight, respectively. Only selected models (i.e., ΔAICc < 2) are shown.
**Table S10:** Model comparison regarding whether participants in our survey had experience with each taxonomic group. Several possible predictor variables were tested (see main text for details). ‘+’ represents that the predictor variable was included in models shown. AICc and W stand for models' Akaike information criterion for small sample sizes and their weight, respectively. Only selected models (i.e., ΔAICc < 2) are shown.

## Data Availability

All data and code used in this study are available at https://zenodo.org/records/22764276.

## Appendix 1 Survey

[Participant Information Sheet and Consent was applied before the survey]

*PART 1: Eligibility*

1. **Have you ever directly collected behavioural data from non‐human organisms that led to at least one research output* of which you were an author?**

*Research outputs include conference posters or presentations, preprints, peer‐reviewed publications, dissertations, theses or books

○ Yes [proceed]
○ No [terminate the survey]

*PART 2: Demographics*

2 **How old are you?**

___________________________________

3 **How do you identify?**

○ Agender
○ Gender fluid
○ Man
○ Non binary
○ Woman
○ Other (please specify)

___________________________________

4 **In which country have you spent most of your life UNTIL you were 18 years old? (please elect only one country if multiple countries apply)**

___________________________________

5 **In which country have you spent most of your life AFTER turning 18 years old? (please elect only one country if multiple countries apply)**

___________________________________

6 **What is your current highest level of education? (i.e., obtained degree; do NOT consider current studies)**

○ High school/Technical school
○ Undergraduate/Honours degree
○ MSc/Master's degree
○ PhD/Doctoral degree

7 **[if Q6 is PhD/Doctoral degree] In what year did you complete your PhD?**

___________________________________

*PART 3: General preferences and experience*

8 **Are you or have you ever been an editor of any scientific journals that at least occasionally publish ethological or behavioural research?**

○ Yes
○ No

9 **Based purely on personal enjoyment, how would you split your time collecting behavioural data between the field and the lab?**

○ 100% in the field, 0% in the lab
○ 90% in the field, 10% in the lab
○ 80% in the field, 20% in the lab
○ 70% in the field, 30% in the lab
○ 60% in the field, 40% in the lab
○ 50% in the field, 50% in the lab
○ 40% in the field, 60% in the lab
○ 30% in the field, 70% in the lab
○ 20% in the field, 80% in the lab
○ 10% in the field, 90% in the lab
○ 0% in the field, 100% in the lab

10 **How much does your desire to work with certain taxonomic groups drive your behavioural research interests compared with other possible motivations (e.g., exploration of specific topics pertinent to most organisms, professional prospects, etc.)?**

○ 100% driven by certain taxonomic group(s), 0% driven by other motivations
○ 90% driven by certain taxonomic group(s), 10% driven by other motivations
○ 80% driven by certain taxonomic group(s), 20% driven by other motivations
○ 70% driven by certain taxonomic group(s), 30% driven by other motivations
○ 60% driven by certain taxonomic group(s), 40% driven by other motivations
○ 50% driven by certain taxonomic group(s), 50% driven by other motivations
○ 40% driven by certain taxonomic group(s), 60% driven by other motivations
○ 30% driven by certain taxonomic group(s), 70% driven by other motivations
○ 20% driven by certain taxonomic group(s), 80% driven by other motivations
○ 10% driven by certain taxonomic group(s), 90% driven by other motivations
○ 0% driven by certain taxonomic group(s), 100% driven by other motivations

11 **How would you rate your feelings towards the following animals on a spectrum ranging from negative (e.g., anxiety, disgust, fear) to positive (e.g., admiration, cuteness, fascination), with ambivalent feelings in between?**

**Table:**

Taxonomic group	Extremely negative	Moderately negative	Slightly negative	Neither positive nor negative	Slightly positive	Moderately positive	Extremely positive
Amphibians							
Arachnids							
Birds							
Crustaceans							
Fish							
Insects							
Mammals							
Molluscs							
Reptiles							

12 **From how many different non‐human organisms (at the species level) have you directly collected behavioural data that led to at least one research output* for which you were an author?**

*Research outputs include conference posters or presentations, preprints, peer‐reviewed publications, dissertations, theses or books.

___________________________________

13 **Select all taxonomic groups from which you directly collected behavioural data that led to at least one research output* of which you were an author**.

*Research outputs include conference posters or presentations, preprints, peer‐reviewed publications, dissertations, theses or books.

○ Amphibian
○ Arachnid
○ Bird
○ Crustacean
○ Fish
○ Insect
○ Invertebrate not on this list
○ Mammal
○ Mollusc.
○ Non‐animal organism
○ Reptile

*PART* *4: Experiences and opinions regarding specific studied organisms*

If Q12 answer is equal to or greater than 2, some of the following questions (Q15–18 and Q27, 28) will be asked twice. However, repeated questions will be related to the species that the participant worked with least recently (see Q14).

14 **Specify the scientific name (genus and epithet, e.g., *Homo sapiens* ) of the [most/least] recent non‐human species from which you directly collected behavioural data that led to at least one research output* of which you were an author (elect one species if multiple apply)**.

*Research outputs include conference posters or presentations, preprints, peer‐reviewed publications, dissertations, theses or books.

___________________________________

15 **[Q14 answer] is a/an**

○ Amphibian
○ Arachnid
○ Bird
○ Crustacean
○ Fish
○ Insect
○ Invertebrate not on this list
○ Mammal
○ Mollusc
○ Non‐animal organism
○ Reptile

16 **Approximately how many peer‐reviewed articles have you published involving behavioural data that you directly collected from [Q14 answer]?**

○ 0
○ 1
○ 2
○ 3
○ 4 or more

17 **When did you first directly collect behavioural data from [Q14 answer]?**

○ During or before my PhD
○ After my PhD, but not while holding a permanent research position (e.g., unemployed, postdoc)
○ After my PhD, while holding a permanent research position (e.g., lecturer, professor)

18 **Which** of **these situations best describes how you INITIALLY decided to work with [Q14 answer]?**

○ My employer, supervisor or collaborator used this species for research and/or suggested me to use this species in my research
○ Someone, but NOT my employer, supervisor or collaborator, showed or told me about this species and/or suggested that I use it in my research
○ I had the idea to use this species in my research before anyone else directly suggested it or influenced me to do it

Questions below are answered on a 7‐point Likert scale (i.e., strongly disagree to strongly agree):

19 **Reaching the place where [Q14 answer] individuals are expected to be, and then locating or attracting them in the field is easy/accessible**.
20 **Once [Q14 answer] individuals are located or attracted, observing or recording their behaviours in the field is easy/accessible**.
21 **Obtaining [Q14 answer] individuals from suppliers (e.g., other research teams, farms, fisheries, pet stores) is easy/accessible**.
22 **Rearing and maintaining [Q14 answer] individuals in the lab is easy/accessible**
23 **Observing or recording [Q14 answer] individuals in the lab is easy/accessible**
24 **[Q14 answer] is considered a model organism by the scientific community**
25 **The behavioural literature on [Q14 answer] is extensive**.
26 **[Q14 answer] possesses a specific characteristic that makes it an ideal species to answer a question I am or was interested in**
27 **I already had the hobby of going out specifically to search for or look at [Q15 answer]s before I started working with [Q14 answer]**
28 **My interest for [Q15 answer]s increased after working with [Q14 answer]**.
29 **[Q14 answer] is an economically important species (e.g., used commercially, provides ecological services, considered a pest or a disease vector)**
30 **[Q14 answer] is endangered/threatened in the wild**
31 **The behavioural research I have conducted with [Q14 answer] can be considered applied (e.g., pest control, conservation)**
32 **Please select the option ‘agree’ [attention question]**

## Appendix 2 Additional Discussion

Justifying the choice of study species has become a staple in research manuscripts (Mann 2015), which may explain why most surveyed researchers agreed that the species they most recently worked with possesses a specific characteristic that makes it ideal to explore a certain topic. However, it is possible that other species could be equally useful for addressing the same questions, as similar traits often occur across diverse taxa, including (apparently) unique characteristics. For example, biologists would intuitively use mammals to understand the evolution of milk‐like secretions as a form of parental care (Clutton‐Brock 1991), but species of other taxonomic groups can also express this behaviour (e.g., cecilians: Mailho‐Fontana et al. 2024; cockroaches: Stay and Coop 1974; spiders: Chen et al. 2018). We thus argue that we could discover more species with astonishing features by exploring the existent biodiversity, which would grant us new ideal organisms to study, instead of simply marketing a few species as ideal for a topic (especially those that have been investigated for decades by several people).

Contradictory results we found, such that preference for fieldwork was positively related to fondness for arachnids but negatively related to the likelihood of working with them, may indicate that researchers are unaware that they can use neglected taxa to pursue their preferences (e.g., work with arachnids in the field), indicating that a diverse taxonomic education is key to addressing taxonomic bias in ecology and evolution. At finer taxonomic levels, participants with a stronger preference for fieldwork were more likely to report that the species they most recently worked with is easy to observe in the field, but harder to work with in the laboratory. This suggests that researchers select specific organisms to match their professional preferences also within a taxonomic group.

We also investigated the relationship between age and gender on researchers' taxonomic preferences and experiences, even though we had no hypotheses on how these variables relate to taxonomic bias. We found that, on average, older participants in our survey worked with more species and taxonomic groups than their younger counterparts, which may simply reflect that they had more time to do so. However, it is also possible that researchers have been recently using fewer taxa in their research, making their experiences less taxonomically diverse compared with researchers in previous decades. We also observed that increasing age was related to greater fondness for birds, lower fondness for amphibians, and greater likelihood of having worked with mammals, which might show that distinct generations of researchers have different perspectives. Women that participated in our survey worked with fewer species and taxonomic groups than men, possibly reflecting the difficulties women face in academia (Casad et al. 2021; Spoon et al. 2023) if these obstacles limit their opportunities for taxonomic diversification. Although the literature suggests that women are more likely to have animal phobias than men (Fredrikson et al. 1996), we found no gender differences in the perception of different taxonomic groups, except for mammals, which women viewed more favourably than men (Figure 3A).

In our survey, we also verified the relationship between participants' editorial experience and their taxonomic focus. Given that editors are the main gatekeepers in the publishing system, they have more power than other individuals to change patterns of taxonomic bias in the literature. Participants with editorial experience worked with more species but not with more taxonomic groups than other participants (Figure 2), showing that these individuals are not specifically selected because of the diversity of their experiences at a high taxonomic level. Despite participants with editorial experience having a more favourable perception of some animal groups (crustaceans, fish, molluscs) than others, they were more likely to work with birds than their counterparts. If editors are more likely to accept research manuscripts that match their personal experiences for publication, then taxonomic bias in the literature can worsen. This is certainly possible as biases in the publication process within ecology and evolution have been documented (Fox et al. 2023; Srivastava et al. 2024), highlighting the need for greater transparency in editorial decisions to assess whether individual preferences and experiences influence publication patterns.
