# Supplementary material for: Drivers of Taxonomic Bias in Ecology and Evolution: Insights From Ethologists and Behavioural Ecologists
Source: Ecol Evol. 2026 Oct 12;16(10):e74460. doi: 10.1002/ece3.74460 (PMC13658930; doi:10.1002/ece3.74460)
Supplement: Supplementary file 1 [file ECE3-16-e74460-s001.docx]

# Supplementary figures

## Figure S1.

Participants’ country. Country in which participants spent most of their life before (A) and after (B) being 18 years old. No participants mentioned countries or regions in grey.

## Figure S2.

Participants’ preferences, motivations, and overall experiences. Proportion of participants expressing their preference for fieldwork over lab work (A) and their motivation from taxa over other factors (B). Proportion of participants that experienced (i.e. directly collected behavioural data from, leading to at least one research output of which they were an author) distinct numbers of species (C), and of taxonomic groups (D).

## Figure S3.

Taxonomic groups that participants experienced (i.e. directly collected behavioural data from, leading to at least one research output of which they were an author). Note that each participant could experience multiple taxa.

## Figure S4.

Taxonomic groups of specific species that participants most and least recently experienced (i.e. directly collected behavioural data from, leading to at least one research output of which they were an author).

## Figure S5.

Participants’ feelings towards distinct animal groups. Responses from all participants together (A) or by experience with the taxon in question (B: without, C: with). In panel A, distinct letters on the right-side of bars represent statistical differences between taxonomic groups within that panel (z-values with p < 0.05 for all pairwise comparisons). In panels B and C, numbers on the right-side of bars represent the number of participants for each taxon in the subset presented, with a total of 868 for each taxon.

# Supplementary tables

## Table S1.

Journals used in our study to retrieve researchers’ emails. ISSN stands for International Standard Serial Number.

| **Publication period** | **Journal** | **Publisher** | **Supporting society or institution** | **ISSN(s)** |
| --- | --- | --- | --- | --- |
| January 2015 – June 2026 | Acta Ethologica | Springer Nature | Portuguese Ethological Society | 0873-9749, 1437-9546 |
|  | Animal Behaviour | Elsevier | Association for the Study of Animal Behaviour, Animal Behavior Society | 1095-8282, 0003-3472 |
|  | Animal Cognition | Springer Nature | - | 1435-9448, 1435-9456 |
|  | Behavioral Ecology | Oxford University Press | International Society for Behavioral Ecology | 1045-2249, 1465-7279 |
|  | Behavioral Ecology and Sociobiology | Springer Nature | - | 0340-5443, 1432-0762 |
|  | Behaviour | Brill | - | 0005-7959, 1568-539X |
|  | Behavioural Processes | Elsevier | - | 0376-6357, 1872-8308 |
|  | Ethology | Wiley | Ethological Society | 0179-1613, 1439-0310 |
|  | Ethology Ecology and Evolution | Taylor & Francis | - | 0394-9370, 1120-6705, 1828-7131, |
|  | Journal of Comparative Psychology | American Psychological Association | - | 0735-7036, 1939-2087 |
|  | Journal of Ethology | Springer Nature | Japan Ethological Society | 0289-0771, 1439-5444 |
| January 2020 – July 2026 | BMC Ecology and Evolution | BMC | - | 2730-7182 |
|  | Current Zoology | Oxford University Press | China Zoological Society | 1674-5507, 2396-9814 |
|  | Ecology and Evolution | Wiley | British Ecological Society | 2045-7758 |
|  | Ecology Letters | Wiley | Centre National de la Recherche Scientifique | 1461-023X, 1461-0248 |
|  | Evolution | Oxford University Press | Society for the Study of Evolution | 0014-3820,  1558-5646 |
|  | Evolution Letters | Oxford University Press | Society for the Study of Evolution | 2056-3744 |
|  | Functional Ecology | Wiley | British Ecological Society | 0269-8463,  1365-2435 |
|  | Journal of Animal Ecology | Wiley | British Ecological Society | 0021-8790,  1365-2656 |
|  | Journal of Evolutionary Biology | Oxford University Press | European Society for Evolutionary Biology | 1010-061X,  1420-9101 |
|  | Nature Ecology and Evolution | Springer Nature | - | 2397-334X |
|  | The American Naturalist | The University of Chicago Press | American Society of Naturalists | 0003-0147, 1537-5323 |

## Table S2.

Model comparison regarding the number of species experienced by participants in our survey. Several possible predictor variables were tested (see main text for details). “+” represents that the predictor variable was included in models shown. *AICc* and *W* stand for models’ Akaike information criterion for small sample sizes and their weight, respectively. Only selected models (i.e. *ΔAICc* < 2) are shown.

| **Rank** | **Predictor variables** | | | | | | ***AICc*** | ***ΔAICc*** | ***W*** |
| --- | --- | --- | --- | --- | --- | --- | --- | --- | --- |
|  | **Number of taxonomic groups experienced** | **Age** | **Gender** | **Editorial experience** | **Preference for fieldwork** | **Motivation from taxa** |  |  |  |
| 1 | + | + | + | + |  | + | 5510.72 | 0.00 | 0.50 |
| 2 | + | + | + | + | + | + | 5512.45 | 1.73 | 0.21 |

## Table S3.

Model comparison regarding the number of taxonomic groups experienced by participants in our survey. Several possible predictor variables were tested (see main text for details). “+” represents that the predictor variable was included in models shown. *AICc* and *W* stand for models’ Akaike information criterion for small sample sizes and their weight, respectively. Only selected models (i.e. *ΔAICc* < 2) are shown.

| **Rank** | **Predictor variables** | | | | | | ***AICc*** | ***ΔAICc*** | ***W*** |
| --- | --- | --- | --- | --- | --- | --- | --- | --- | --- |
|  | **Number of species experienced** | **Age** | **Gender** | **Editorial experience** | **Preference for**  **fieldwork** | **Motivation from taxa** |  |  |  |
| 1 | + | + |  | + |  | + | 2681.64 | 0.00 | 0.25 |
| 2 | + | + |  |  |  | + | 2682.02 | 0.39 | 0.21 |
| 3 | + | + |  | + | + | + | 2683.16 | 1.53 | 0.12 |
| 4 | + | + | + | + |  | + | 2683.16 | 1.53 | 0.12 |
| 5 | + | + | + |  |  | + | 2683.49 | 1.86 | 0.10 |
| 6 | + | + |  |  | + | + | 2683.59 | 1.96 | 0.09 |

## Table S4.

Model comparison regarding five statements (response variables) about the ease to work with the most recent species experienced by participants in our survey. “+” represents that the predictor variable was included in models shown. *AICc* and *W* stand for models’ Akaike information criterion for small sample sizes and their weight, respectively.

| **Response variable** | **Rank** | **Predictor variables** | | ***AICc*** | ***ΔAICc*** | ***W*** |
| --- | --- | --- | --- | --- | --- | --- |
|  |  | **Genus mentions** | **Preference for fieldwork** |  |  |  |
| Easy to find in the field | 1 | + |  | 3112.52 | 0.00 | 0.70 |
|  | 2 | + | + | 3114.45 | 1.92 | 0.27 |
|  | 3 |  |  | 3119.07 | 6.55 | 0.03 |
|  | 4 |  | + | 3120.6 | 8.08 | 0.01 |
| Easy to observe in the field | 1 | + | + | 3139.08 | 0.00 | 0.93 |
|  | 2 |  | + | 3144.53 | 5.44 | 0.06 |
|  | 3 | + |  | 3148.09 | 9.00 | 0.01 |
|  | 4 |  |  | 3151.57 | 12.49 | 0.00 |
| Easy to rear in the lab | 1 | + | + | 2675.73 | 0.00 | 1.00 |
|  | 2 |  | + | 2709.14 | 33.41 | 0.00 |
|  | 3 | + |  | 2717.62 | 41.89 | 0.00 |
|  | 4 |  |  | 2753.52 | 77.8 | 0.00 |
| Easy to observe in the lab | 1 | + | + | 2631.58 | 0.00 | 1.00 |
|  | 2 |  | + | 2674.9 | 43.32 | 0.00 |
|  | 3 | + |  | 2675.63 | 44.05 | 0.00 |
|  | 4 |  |  | 2721.03 | 89.45 | 0.00 |
| Easy to buy | 1 | + |  | 2182.53 | 0.00 | 1.00 |
|  | 2 | + | + | 2193.97 | 11.43 | 0.00 |
|  | 3 |  |  | 2219.78 | 37.25 | 0.00 |
|  | 4 |  | + | 2234.16 | 51.63 | 0.00 |
|  |  |  |  |  |  |  |

## Table S5.

Coefficients from models selected regarding five statements (response variables) about the ease to work with the most recent species experienced by participants in our survey (see also Table S4).

| **Response variable** | **Predictor variable** | **Coefficient** | **SE** | **z-score** | **p-value** |
| --- | --- | --- | --- | --- | --- |
| Easy to find in the field | Genus mentions | 0.14 | 0.05 | 2.90 | <0.001 |
| Easy to observe in the field | Genus mentions | 0.11 | 0.04 | 2.68 | 0.01 |
|  | Preference for fieldwork | 0.24 | 0.07 | 3.33 | <0.001 |
| Easy to rear in the lab | Genus mentions | 0.35 | 0.06 | 5.92 | <0.001 |
|  | Preference for fieldwork | -0.57 | 0.09 | -6.63 | <0.001 |
| Easy to observe in the lab | Genus mentions | 0.39 | 0.06 | 6.64 | <0.001 |
|  | Preference for fieldwork | -0.58 | 0.09 | -6.76 | <0.001 |
| Easy to buy | Genus mentions | 0.45 | 0.07 | 6.02 | <0.001 |
|  | Preference for fieldwork | -0.35 | 0.10 | -3.68 | <0.001 |

## Table S6.

Model comparison regarding four statements (response variables) about the most recent species experienced by participants in our survey. “+” represents that the predictor variable was included in models shown. *AICc* and *W* stand for models’ Akaike information criterion for small sample sizes and their weight, respectively.

| **Response variable** | **Rank** | **Predictor variable** | ***AICc*** | ***ΔAICc*** | ***W*** |
| --- | --- | --- | --- | --- | --- |
|  |  | **Genus mentions** |  |  |  |
| Possess a specific characteristic that makes it ideal to answer a question | 1 | + | 2092.56 | 0.00 | 0.98 |
|  | 2 |  | 2100.38 | 7.83 | 0.02 |
| Economically important | 1 | + | 2958.34 | 0.00 | 0.90 |
|  | 2 |  | 2962.75 | 4.41 | 0.10 |
| Endangered or threatened in the wild | 1 | + | 2581.36 | 0.00 | 1.00 |
|  | 2 |  | 2592.29 | 10.94 | 0.00 |
| Considered a model organism by the scientific community | 1 | + | 2928.52 | 0.00 | 1.00 |
|  | 2 |  | 3021.65 | 93.13 | 0.00 |

## Table S7.

Coefficients from models selected regarding four statements (response variables) about the most recent species experienced by participants in our survey (see also Table S6).

| **Response variable** | **Predictor variable** | **Coefficient** | **SE** | **z-score** | **p-value** |
| --- | --- | --- | --- | --- | --- |
| Possess a specific characteristic that makes it ideal to answer a question | Genus mentions | 0.12 | 0.04 | 3.18 | <0.001 |
| Economically important | Genus mentions | 0.16 | 0.06 | 2.51 | 0.01 |
| Endangered or threatened in the wild | Genus mentions | -0.24 | 0.07 | -3.62 | <0.001 |
| Considered a model organism by the scientific community | Genus mentions | 0.54 | 0.06 | 9.73 | <0.001 |

## Table S8.

Model comparison regarding whether the species mentioned by participants in our survey were suggested by a supervisor (*vs.* other people or themselves). Two possible predictor variables were tested (see main text for details). “+” represents that the predictor variable was included in models shown. *AICc* and *W* stand for models’ Akaike information criterion for small sample sizes and their weight, respectively.

| **Rank** | **Predictor variables** | | ***AICc*** | ***ΔAICc*** | ***W*** |
| --- | --- | --- | --- | --- | --- |
|  | **Period** | **Version** |  |  |  |
| 1 | + |  | 1871.87 | 0.00 | 0.61 |
| 2 | + | + | 1872.73 | 0.86 | 0.39 |
| 3 |  | + | 2013.53 | 141.66 | 0.00 |
| 4 |  |  | 2042.80 | 170.93 | 0.00 |

## Table S9.

Model comparison regarding the fondness by participants in our survey for each taxonomic group. Several possible predictor variables were tested (see main text for details). “+” represents that the predictor variable was included in in models shown. *AICc* and *W* stand for models’ Akaike information criterion for small sample sizes and their weight, respectively. Only selected models (i.e. *ΔAICc* < 2) are shown.

| **Taxon** | **Rank** | **Predictor variables** | | | | | | | ***AICc*** | ***ΔAICc*** | ***W*** |
| --- | --- | --- | --- | --- | --- | --- | --- | --- | --- | --- | --- |
|  |  | **Experience** | **Number of taxonomic groups experienced** | **Age** | **Gender** | **Editorial experience** | **Preference for fieldwork** | **Motivation from taxa** |  |  |  |
| Amphibian | 1 | + |  |  |  | + | + |  | 2186.14 | 0.00 | 0.09 |
|  | 2 | + |  |  | + | + | + |  | 2186.35 | 0.21 | 0.08 |
|  | 3 | + |  |  |  |  | + |  | 2186.40 | 0.26 | 0.08 |
|  | 4 | + |  | + |  | + | + |  | 2186.58 | 0.44 | 0.07 |
|  | 5 | + | + |  | + | + | + |  | 2187.30 | 1.16 | 0.05 |
|  | 6 | + |  |  | + |  | + |  | 2187.38 | 1.24 | 0.05 |
|  | 7 | + | + |  |  |  | + |  | 2187.44 | 1.30 | 0.05 |
|  | 8 | + | + |  |  | + | + |  | 2187.49 | 1.35 | 0.04 |
|  | 9 | + | + | + |  | + | + |  | 2187.51 | 1.37 | 0.04 |
|  | 10 | + |  | + | + | + | + |  | 2187.55 | 1.41 | 0.04 |
|  | 11 | + | + |  | + |  | + |  | 2187.94 | 1.81 | 0.03 |
|  | 12 | + |  |  |  | + | + | + | 2188.10 | 1.97 | 0.03 |
| Arachnid | 1 | + |  | + |  |  | + | + | 2958.94 | 0.00 | 0.08 |
|  | 2 | + |  | + |  |  | + |  | 2959.60 | 0.66 | 0.06 |
|  | 3 | + |  |  |  |  | + | + | 2960.18 | 1.24 | 0.04 |
|  | 4 | + |  |  |  | + | + | + | 2960.51 | 1.57 | 0.04 |
|  | 5 | + |  | + |  | + | + | + | 2960.61 | 1.67 | 0.04 |
|  | 6 | + | + | + |  |  | + | + | 2960.67 | 1.74 | 0.03 |
|  | 7 | + | + | + |  |  | + |  | 2960.74 | 1.8 | 0.03 |
|  | 8 | + |  |  |  |  | + |  | 2960.78 | 1.84 | 0.03 |
|  | 9 | + |  | + | + |  | + | + | 2960.78 | 1.85 | 0.03 |
| Bird | 1 | + | + | + |  |  | + |  | 1846.31 | 0.00 | 0.26 |
|  | 2 | + | + | + | + |  | + |  | 1846.94 | 0.63 | 0.19 |
|  | 3 | + | + | + |  | + | + |  | 1847.96 | 1.65 | 0.11 |
| Crustacean | 1 | + |  |  |  | + |  |  | 2544.91 | 0 | 0.14 |
|  | 2 | + |  |  |  | + |  | + | 2545.31 | 0.4 | 0.12 |
|  | 3 | + | + |  |  | + |  |  | 2545.65 | 0.74 | 0.10 |
|  | 4 | + | + |  |  | + |  | + | 2546.59 | 1.68 | 0.06 |
|  | 5 | + |  | + |  | + |  |  | 2546.85 | 1.94 | 0.05 |
| Fish | 1 | + |  |  |  | + |  |  | 2399.70 | 0.00 | 0.10 |
|  | 2 | + |  |  |  | + | + |  | 2399.97 | 0.28 | 0.09 |
|  | 3 | + |  | + |  | + |  |  | 2400.10 | 0.40 | 0.08 |
|  | 4 | + |  | + |  | + | + |  | 2400.50 | 0.80 | 0.07 |
|  | 5 | + |  |  |  | + | + | + | 2400.97 | 1.27 | 0.05 |
|  | 6 | + |  |  |  | + |  | + | 2401.11 | 1.41 | 0.05 |
|  | 7 | + | + |  |  | + |  |  | 2401.39 | 1.69 | 0.04 |
|  | 8 | + |  | + |  | + | + | + | 2401.47 | 1.77 | 0.04 |
|  | 9 | + |  | + |  | + |  | + | 2401.48 | 1.78 | 0.04 |
|  | 10 | + | + | + |  | + |  |  | 2401.51 | 1.82 | 0.04 |
|  | 11 | + | + |  |  | + | + |  | 2401.62 | 1.92 | 0.04 |
| Insect | 1 | + |  |  |  | + |  |  | 2425.11 | 0.00 | 0.07 |
|  | 2 | + |  |  |  |  |  |  | 2425.51 | 0.40 | 0.05 |
|  | 3 | + | + |  |  | + |  |  | 2425.64 | 0.52 | 0.05 |
|  | 4 | + |  |  |  | + | + |  | 2426.12 | 1.01 | 0.04 |
|  | 5 | + |  |  |  |  | + |  | 2426.49 | 1.38 | 0.03 |
|  | 6 | + | + |  |  | + | + |  | 2426.50 | 1.39 | 0.03 |
|  | 7 | + | + |  |  |  |  |  | 2426.55 | 1.43 | 0.03 |
|  | 8 | + |  | + |  |  |  |  | 2426.79 | 1.67 | 0.03 |
|  | 9 | + |  |  | + |  |  |  | 2426.88 | 1.77 | 0.03 |
|  | 10 | + |  |  | + | + |  |  | 2426.96 | 1.85 | 0.03 |
|  | 11 | + |  |  |  | + |  | + | 2427.04 | 1.93 | 0.03 |
|  | 12 | + |  | + |  | + |  |  | 2427.08 | 1.96 | 0.02 |
| Mammal | 1 | + | + |  | + |  | + |  | 1812.87 | 0.00 | 0.29 |
|  | 2 | + | + |  | + | + | + |  | 1813.39 | 0.51 | 0.22 |
|  | 3 | + | + | + | + |  | + |  | 1814.46 | 1.58 | 0.13 |
| Mollusc | 1 | + |  |  |  | + |  |  | 2629.47 | 0.00 | 0.09 |
|  | 2 | + |  |  | + | + |  |  | 2630.14 | 0.67 | 0.06 |
|  | 3 | + |  |  |  | + |  | + | 2630.27 | 0.80 | 0.06 |
|  | 4 | + | + |  |  | + |  |  | 2630.31 | 0.84 | 0.06 |
|  | 5 | + |  |  | + | + |  | + | 2630.70 | 1.23 | 0.05 |
|  | 6 | + |  |  |  | + | + |  | 2630.88 | 1.41 | 0.04 |
|  | 7 | + | + |  | + | + |  |  | 2631.02 | 1.54 | 0.04 |
|  | 8 | + |  |  |  | + | + | + | 2631.36 | 1.89 | 0.03 |
| Reptile | 1 | + |  | + |  |  | + |  | 2343.39 | 0.00 | 0.22 |
|  | 2 | + |  | + |  | + | + |  | 2344.82 | 1.43 | 0.11 |
|  | 3 | + |  | + | + |  | + |  | 2345.21 | 1.82 | 0.09 |
|  | 4 | + |  | + |  |  | + | + | 2345.34 | 1.95 | 0.08 |

## Table S10.

Model comparison regarding whether participants in our survey had experience with each taxonomic group. Several possible predictor variables were tested (see main text for details). “+” represents that the predictor variable was included in in models shown. *AICc* and *W* stand for models’ Akaike information criterion for small sample sizes and their weight, respectively. Only selected models (i.e. *ΔAICc* < 2) are shown.

| **Taxon** | **Rank** | **Predictor variables** | | | | | | | ***AICc*** | ***ΔAICc*** | ***W*** |
| --- | --- | --- | --- | --- | --- | --- | --- | --- | --- | --- | --- |
|  |  | **Fondness** | **Number of taxonomic groups experienced** | **Age** | **Gender** | **Editorial experience** | **Preference for fieldwork** | **Motivation from taxa** |  |  |  |
| Amphibian | 1 | + | + | + |  |  |  | + | 504.54 | 0.00 | 0.28 |
|  | 2 | + | + | + |  | + |  | + | 506.13 | 1.59 | 0.12 |
|  | 3 | + | + |  |  |  |  | + | 506.37 | 1.83 | 0.11 |
|  | 4 | + | + | + |  |  | + | + | 506.43 | 1.89 | 0.11 |
| Arachnid | 1 | + | + | + | + |  | + | + | 463.99 | 0.00 | 0.17 |
|  | 2 | + | + | + |  |  | + | + | 464.48 | 0.49 | 0.13 |
|  | 3 | + | + | + | + | + | + | + | 464.81 | 0.82 | 0.11 |
|  | 4 | + | + |  |  | + | + | + | 464.99 | 1.00 | 0.10 |
|  | 5 | + | + |  | + | + | + | + | 465.30 | 1.31 | 0.09 |
|  | 6 | + | + | + |  | + | + | + | 465.39 | 1.40 | 0.08 |
|  | 7 | + | + |  |  |  | + | + | 465.56 | 1.58 | 0.08 |
| Bird | 1 | + | + | + |  | + | + |  | 969.83 | 0.00 | 0.28 |
|  | 2 | + | + |  |  | + | + |  | 970.79 | 0.97 | 0.17 |
|  | 3 | + | + | + |  | + | + | + | 971.43 | 1.60 | 0.13 |
| Crustacean | 1 | + | + |  |  | + | + |  | 385.54 | 0.00 | 0.19 |
|  | 2 | + | + |  |  |  | + |  | 386.71 | 1.17 | 0.11 |
|  | 3 | + | + |  |  | + | + | + | 387.00 | 1.47 | 0.09 |
|  | 4 | + | + | + |  | + | + |  | 387.39 | 1.86 | 0.08 |
| Fish | 1 | + | + | + |  |  | + |  | 732.05 | 0.00 | 0.15 |
|  | 2 | + | + | + | + |  | + |  | 732.49 | 0.44 | 0.12 |
|  | 3 | + | + | + |  | + | + |  | 732.79 | 0.73 | 0.10 |
|  | 4 | + | + |  | + | + | + |  | 732.8 | 0.74 | 0.10 |
|  | 5 | + | + | + | + | + | + |  | 733.27 | 1.21 | 0.08 |
|  | 6 | + | + |  |  | + | + |  | 733.52 | 1.47 | 0.07 |
|  | 7 | + | + |  | + |  | + |  | 733.64 | 1.59 | 0.07 |
|  | 8 | + | + | + |  |  | + | + | 733.94 | 1.88 | 0.06 |
| Insect | 1 | + | + |  |  |  | + |  | 868.92 | 0.00 | 0.19 |
|  | 2 | + | + |  |  |  | + | + | 869.39 | 0.47 | 0.15 |
|  | 3 | + | + | + |  |  | + |  | 869.64 | 0.72 | 0.13 |
|  | 4 | + | + |  |  | + | + |  | 870.03 | 1.12 | 0.11 |
|  | 5 | + | + | + |  |  | + | + | 870.28 | 1.37 | 0.09 |
|  | 6 | + | + |  |  | + | + | + | 870.56 | 1.64 | 0.08 |
| Mammal | 1 | + | + | + | + |  | + |  | 1057.46 | 0.00 | 0.2 |
|  | 2 | + | + | + |  |  | + |  | 1058.16 | 0.7 | 0.14 |
|  | 3 | + | + | + | + |  | + | + | 1058.31 | 0.85 | 0.13 |
|  | 4 | + | + | + | + | + | + |  | 1058.61 | 1.15 | 0.11 |
|  | 5 | + | + | + |  |  | + | + | 1058.76 | 1.3 | 0.1 |
|  | 6 | + | + | + |  | + | + |  | 1059.36 | 1.90 | 0.08 |
| Mollusc | 1 | + | + |  |  |  | + |  | 318.33 | 0.00 | 0.12 |
|  | 2 | + | + | + |  | + | + |  | 318.68 | 0.36 | 0.10 |
|  | 3 | + | + |  |  | + | + |  | 318.74 | 0.41 | 0.10 |
|  | 4 | + | + | + |  |  | + |  | 319.48 | 1.16 | 0.07 |
|  | 5 | + | + |  |  |  |  |  | 319.68 | 1.35 | 0.06 |
|  | 6 | + | + |  |  |  | + | + | 319.81 | 1.48 | 0.06 |
|  | 7 | + | + | + |  | + |  |  | 320.06 | 1.73 | 0.05 |
|  | 8 | + | + |  |  | + |  |  | 320.10 | 1.77 | 0.05 |
| Reptile | 1 | + | + |  |  |  |  |  | 607.85 | 0.00 | 0.14 |
|  | 2 | + | + |  |  |  | + |  | 608.20 | 0.35 | 0.12 |
|  | 3 | + | + |  |  | + |  |  | 609.31 | 1.46 | 0.07 |
|  | 4 | + | + |  |  |  |  | + | 609.31 | 1.46 | 0.07 |
|  | 5 | + | + |  |  | + | + |  | 609.75 | 1.90 | 0.06 |
| Other invertebrate | 1 |  | + |  |  |  | + |  | 314.41 | 0.00 | 0.21 |
|  | 2 |  | + |  |  | + | + |  | 314.65 | 0.23 | 0.19 |
|  | 3 |  | + | + |  |  | + |  | 316.20 | 1.79 | 0.09 |
|  | 4 |  | + |  |  |  | + | + | 316.37 | 1.96 | 0.08 |
| Other organism | 1 |  | + |  |  |  |  |  | 211.88 | 0.00 | 0.13 |
|  | 2 |  | + | + |  |  |  |  | 212.14 | 0.25 | 0.11 |
|  | 3 |  | + |  | + |  |  |  | 212.76 | 0.88 | 0.08 |
|  | 4 |  | + | + | + |  |  |  | 213.75 | 1.87 | 0.05 |
|  | 5 |  | + |  |  |  | + |  | 213.77 | 1.89 | 0.05 |
|  | 6 |  | + |  |  | + |  |  | 213.79 | 1.91 | 0.05 |
|  | 7 |  | + |  |  |  |  | + | 213.82 | 1.93 | 0.05 |
